# The neuroanatomy of the siboglinid *Riftia pachyptila* highlights sedentarian annelid nervous system evolution

**DOI:** 10.1371/journal.pone.0198271

**Published:** 2018-12-13

**Authors:** Nadezhda N. Rimskaya-Korsakova, Sergey V. Galkin, Vladimir V. Malakhov

**Affiliations:** 1 Department of Invertebrate Zoology, Faculty of Biology, Lomonosov Moscow State University, Moscow, Russia; 2 Laboratory of Ocean Benthic Fauna, Shirshov Institute of Oceanology of the Russian Academy of Science, Moscow, Russia; 3 Far Eastern Federal University, Vladivostok, Russia; Universitetet i Bergen, NORWAY

## Abstract

Tracing the evolution of the siboglinid group, peculiar group of marine gutless annelids, requires the detailed study of the fragmentarily explored central nervous system of vestimentiferans and other siboglinids. 3D reconstructions of the neuroanatomy of *Riftia* revealed that the “brain” of adult vestimentiferans is a fusion product of the supraesophageal and subesophageal ganglia. The supraesophageal ganglion-like area contains the following neural structures that are homologous to the annelid elements: the peripheral perikarya of the brain lobes, two main transverse commissures, mushroom-like structures, commissural cell cluster, and the circumesophageal connectives with two roots which give rise to the palp neurites. Three pairs of giant perikarya are located in the supraesophageal ganglion, giving rise to the paired giant axons. The circumesophageal connectives run to the VNC. The subesophageal ganglion-like area contains a tripartite ventral aggregation of perikarya (= the postoral ganglion of the VNC) interconnected by the subenteral commissure. The paired VNC is intraepidermal, not ganglionated over most of its length, associated with the ciliary field, and comprises the giant axons. The pairs of VNC and the giant axons fuse posteriorly. Within siboglinids, the vestimentiferans are distinguished by a large and considerably differentiated brain. This reflects the derived development of the tentacle crown. The tentacles of vestimentiferans are homologous to the annelid palps based on their innervation from the dorsal and ventral roots of the circumesophageal connectives. Neuroanatomy of the vestimentiferan brains is close to the brains of Cirratuliiformia and Spionida/Sabellida, which have several transverse commissures, specific position of the giant somata (if any), and palp nerve roots (if any). The palps and palp neurite roots originally developed in all main annelid clades (basally branching, errantian and sedentarian annelids), show the greatest diversity in their number in sedentarian species. Over the course of evolution of Sedentaria, the number of palps and their nerve roots either dramatically increased (as in vestimentiferan siboglinids) or were lost.

## Introduction

Vestimentifera is a peculiar group of marine gutless annelids mainly inhabiting hydrothermal vents and hydrocarbon seeps [1–3]. Vestimentiferan tubeworms, together with Frenulata [4], *Sclerolinum* [5] and the bone-eating worms *Osedax* [6], comprise the annelid group Siboglinidae [7]. The phylogenetic position of Vestimentifera and the whole group Siboglinidae in the annelid system remains controversial. Various annelid sister groups, occuping positions far from each other in the annelid tree, have been proposed, e.g. Oweniidae [8,9], Sabellidae [10,11], Cirratuliformia [12,13], or Clitellata [14,15]. The very peculiar morphology of the vestimentifera and other siboglinids is one reason why their phylogenetic position remains unresolved. Importantly, no comprehensive comparative anatomical study of the organ systems, including neural anatomy, is available to logically favor one of the hypothesized annelid affinities of siboglinids.

The nervous systems of the vestimentiferans and the remaining siboglinids were studied by various methods and different levels of accuracy, making them difficult to compare. Vestimentiferan neuroanatomy was studied in some lamellibrachids (*Lamellibrachia barhami* [4], *L*. *luymesi* [16,17], *L*. *satsuma* [18]) and tevniids (*Riftia pachyptila* [19–21], *Ridgeia piscesae* [22], *Oasisia alvinae* [23]). The ventral nerve cords and brains, including the positions of the perikarya and neuropile, were studied in larvae [24,25] and adults [16,18,21–23,26] using light microscopy and histological techniques. Electron microscopical studies of the neural structures revealed the presence of sensory cells and of glial cells surrounding the neuropile and forming a myelin sheath around the giant axons [21]. The architecture of the frenulate central nervous system is known based on histological and histochemical studies of the ventral nerve cords, neuropile rings and brain area of the supposedly early-branching species *Siboglinum caulleryi*, *S*. *fiordicum* and *Nereilinum murmanicum*, and derived ones such as *Polybrachia annulata and Spirobrachia grandis* [7,27–32]. Electron microscopy revealed the presence of glial and sensory elements in the epidermis of frenulates [33]. The structure of the central nervous system of females and dwarf males of seven species of *Osedax* was described by combining immunohistochemistry with confocal microscopy. This approach revealed numerous commissures and connectives in the brain and trunk nervous system [34,35]. Histological studies of the brain of *Sclerolinum contortum* revealed layers of apical perikarya and a basal neuropile [36]. Nonetheless, our anatomical knowledge of the organization of the nervous system in vestimentiferans and other siboglinids remains fragmentary. Among siboglinids, the neuroanatomy of *Osedax* has been studied in the greatest detail. Reconstructions of the neural architecture of the other siboglinids are therefore crucial in tracing the evolution of the key neuroanatomical features of siboglinids.

We currently lack comparisons of the organizations of the central nervous systems of siboglinids and supposedly closely-related annelid groups [11,21,26,37,38]. First attempts have been made on selected vestimentiferans on smaller sizes [16,18,20] and *Osedax*[34,35]. Miyamoto [18] suggested that the vestimentiferan brain is a simple structure resembling the brain organization of some sedentarian species. The other authors discussed the possible annelid structure of the ventral nerve cord but did not analyze the brain configuration in detail. This restricts the application of the comparative anatomy approach to study the evolution of the siboglinids and annelids.

In the gutless siboglinids the brain occupies an annelid-atypical antero-ventral position. Based on the fact that a coelomic channel passes through the brain, i.e. a rudimentary gut is present in young specimens, Jones and Gardiner [20] suggested that the vestimentiferan brain is a result of the fusion of the supra- and subesophageal ganglia and the circumesophageal connectives. However, fully visualizing the disposition of the transverse commissures of the supraesophageal ganglion and circumesophageal connectives remains to be done. This would highlight not only the brain structure but also help reconstruct the ventral brain formation in vestimentiferans.

Thus, the present study reconstructs the organization of the central nervous system, including the brain and nerve cord, of the large vestimentiferan tubeworm *Riftia pachyptila*. The focus is on brain reconstruction. Among siboglinids, only vestimentiferan juveniles preserve gut rudiments. This makes them a key group to homologize the brain parts of the ventral brain of siboglinids and annelids. The overall aim is to examine the structures of the supra- and subesophageal ganglia and of the circumesophageal connectives in order to provide a comparative neuroanatomical analysis of the vestimentiferans versus other annelid groups. This calls for comprehensive data on the brain and nerve cord structures to determine the ancestral features of the siboglinid nervous system and to reveal the evolution of neural characters within sedentarian annelids.

## Materials and methods

### Collection and fixation

Five specimens of *Riftia pachyptila* Jones, 1981 [19] were collected at different latitudes of the East Pacific Rise (EPR), including the Guaymas Basin, Gulf of California, by the *Pisces* manned submersible during the 12th cruise of the RV Akademik Mstislav Keldysh in 1986 and by *Mir*-1 & 2 manned submersibles during their 49th cruises in 2003. Lengths of examined specimens range from 8 to 808 mm. For data on collection sites, sexes and fixation of specimens see Table 1.

**Table 1:** *Riftia pachyptila* specimens collected during cruises of the RV *Akademik Mstislav Keldysh* (AMK).

specimens				collection sites		
#	sex	length, mm	fixation	name & coordinates	depth, m	# station, ROV, year
**1**	juvenile	8	Bouin’s solution	***9°N EPR***: 09°50,53'N, 104°17,51'W	2552	АМК-4668, *Mir-1*, 2003
**2**	female	16	Bouin’s solution	***Guaymas Basin***: 27°02,45'N, 111°22,80'W	1990	AMK-1519, *Pisces-VII*, 1986
**3**	female	34	Bouin’s solution	***9°N EPR***: 09°50,52'N, 104°17,52'W	2524	АМК-4623, *Mir-1*, 2003
**4**	male	79	Bouin’s solution	***9°N EPR***: 09°50,52'N, 104°17,52'W	2524	АМК-4623, *Mir-1*, 2003
**5**	female	808	formalin	***Guaymas Basin***: 27°00,47'N, 111°24,57'W	2001	АМК-4714, *Mir-2*, 2003

### Histology and LM photography

Animals used for histological analysis were fixed in Bouin’s solution, rinsed three times in 70% ethanol and stored in 70% ethanol prior the histological processing. The material was processed by the standard histological procedure: dehydration in series of alcohol and xylene (80% ethanol, 96% ethanol, mixture of ethanol and buthanol (1:1), buthanol, mixture of buthanol and xylene, xylene (2 changes), 1 hour each change, room temperature), and embedding in paraplast or histowax [39]. Transverse sections (5 and 7 μm) were produced with a Leica RM 2125 microtome (Leica Microsystems, Wetzlar, Germany), stained with Caracci hematoxylin, and examined under a Zeiss Axioplan2 microscope equipped with an AxioCam HRm camera (Carl Zeiss Microscopy, LLC, United States) as well as a Leica DM5000 B microscope equipped with a Leica DFC425 C camera. Microscopic images were optimized for contrast and level using Adobe Photoshop 7.0 (Adobe Systems, San Jose, CA, USA). Drawings were done with Adobe Illustrator CC 2014. To visualize the anastomosing neurites in the trunk epidermis, a 808-mm-long specimen was photographed with a Canon Power Shot S90 camera. For better understanding the trasverse sections, the dorsal sides are always at top. In case of sagittal sections, anterior ends to left.

### 3D modeling

The arrangement of neurite bundles in the brain and the anteriormost ventral nerve cord were visualized with the software 3D-DOCTOR 3.5.040724 (Able Software Corporation of Lexington, USA). Alignment was performed using the same software by comparing the sections of adjacent planes. Image series of 77 cross sections of the 78-mm-long specimen were used tp model the brain organization. 19 objects were traced inside the brain, including the boundary of the brain. Photos were saved in JPEG format with a resolution of 3900 x 3090 pixels and 8 bits / pixel. The field of view was 2812 μm, the parameters of the voxels of the images are 0.721 x 0.721 x 15 μm3. The outlined boundaries yielded 3D models. The implemented smoothing tool was used for natural perception of object surfaces. Interactive features as well as a transparency filter, different colours and lighting effects were applied to show complex and hidden objects. Three-dimensional images under appropriate angles were processed in Adobe Photoshop 7.0.

## Results

### Gross anatomy of nervous system

**Fig 1:**
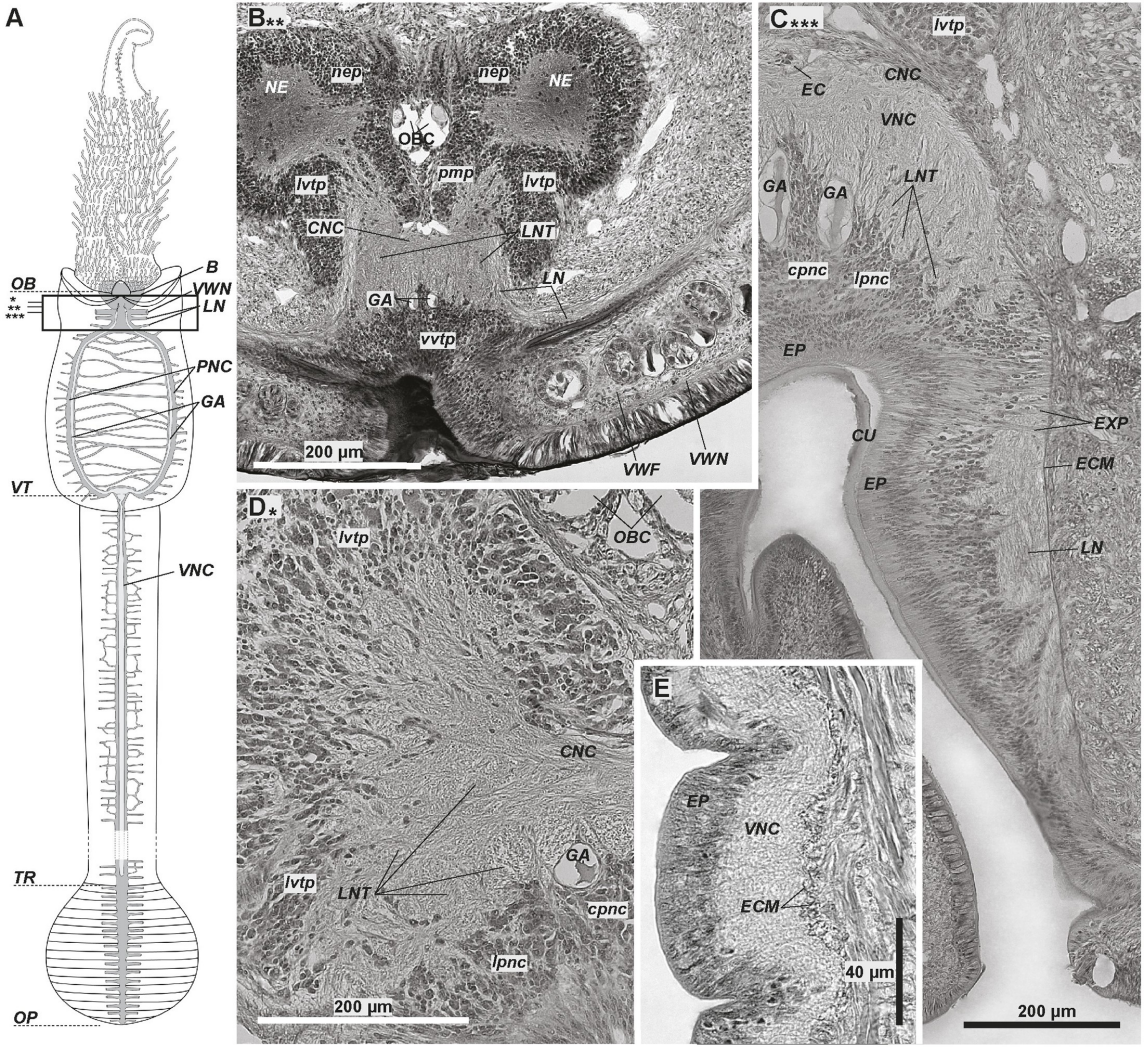
The anterior ventral nerve cord within the vestimental collar of *Riftia pachyptila*. In the anteriormost part of the vestimentum, the ventral brain connects to the ventral nerve cord (A, B). The longitudinal nerve tracts (*LNT)* are continuations of the nerve fibres running from the ventral nerve cord to the posterior and anterior parts of the brain (D, C). The nerve cord lies completely inside the epidermis (E). A—scheme of the central nervous system: main elements in grey, giant axons in light grey. Anterior ends at top. Frame indicates the area corresponding to transverse (B-D) and parasagittal (E) sections. Stars show the levels of the corresponding transverse sections. Parasagittal section (E) made through one of the paired strands of the VNC. Dotted lines show region borders. B—posteriormost brain; elements of ventral nerve cord projecting into brain, juvenile specimen. C–ventral nerve cord (*VNC*) directly posterior to brain, adult specimen. D–longitudinal nerves at transition of ventral nerve cord and brain. E–intraepidermal ventral nerve cord (*VNC*). *B*–brain, *CNC–*commissural neurite bundles of *VNC*, *cpnc–*central perikarya of *VNC*, *CU–*cuticle, *ECM–*extracellular matrix, *EP*–epidermis, *EXP–*epidermal cell processes, *GA*–giant axons, *EC*–enteral coelom, *LN–*circular neurite bundles, *lpnc–*lateral perikarya of VNC, *lvtp–*ventrolateral perikarya of tripartite ventral aggregation, *NE*–neuropile of lateral brain lobes, *nep*–peripheral perikarya of lateral brain lobes, *OB–*obturaculum, *OBC*–obturacular coelom, *OP*–opisthosome, *pmp*–posterior median perikarya aggregation, *PNC–*paired strands of VNC surrounding ventral ciliary field, *vvtp–*ventral perikarya of tripartite ventral aggregation, *TR*–trunk, *VNC–*ventral nerve cord, *VT*–vestimentum, *VWF–*collar of vestimental wings, *VWN–*neurite bundle of *VW*.

**Fig 2:**
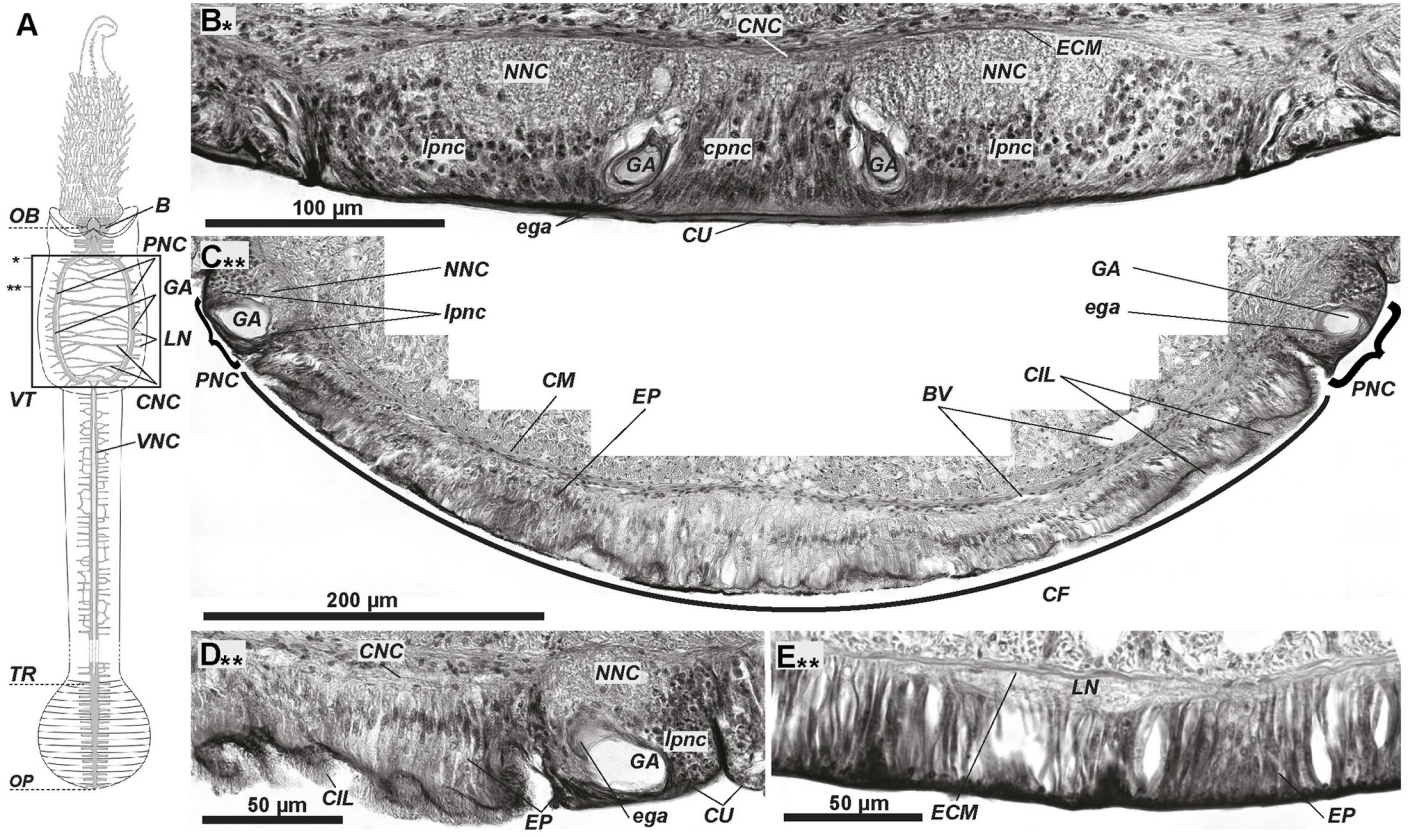
The ventral nerve cord in the vestimentum of *Riftia*. In the vestimentum, the paired strands of the ventral nerve cord (*VNC*) split and surround the ciliary field (*CF*, A-C). The pair of giant axons lies in each strand of the *VNC* (C, D). Numerous lateral neurite bundles arise from the nerve strands (E). The strands and giant axons unite at the boarder between the vestimentum and trunk (A). A–scheme of central nervous system: main elements in grey, giant axons in light grey. Anterior ends at top. Frame indicates area corresponding to histological cross sections (B-E). Stars show the levels of the corresponding sections. B–ventral nerve cord directly anteriorly to ventral ciliary field. C–ventral ciliary field (*CF*) surrounded by paired strands of ventral nerve cord (*PNC*); line shows border of ciliary field, brackets show strands of ventral nerve cord. D–close-up of left strand of ventral nerve cord, commissural neurite bundles connecting the paired strands are visible (*CNC*). E–lateral circular neurite bundles (*LN*) in epidermis. F–one of the lateral neurite bundles running in the body wall epidermis. *B*–brain, *BV–*blood vessels, *CNC–*commissural neurite bundles of *VNC*, *CM*–circular musculature, *CF–*ventral ciliary field, *CIL–*cilia, *cpnc–*central perikarya of *VNC*, *CU–*cuticle, *ECM–*extracellular matrix, *ega–*cells coating *GA*, *EP*–epidermis, *GA*–giant axons, *LN–*circular neurite bundles, *lpnc–*lateral perikarya of *VNC*, *NNC–*neuropile of VNC, *OB–*obturaculum, *OP*–opisthosome, *PNC–*paired strands of VNC surrounding CF, *TR*–trunk, *VNC–*ventral nerve cord, *VT*–vestimentum.

**Fig 3:**
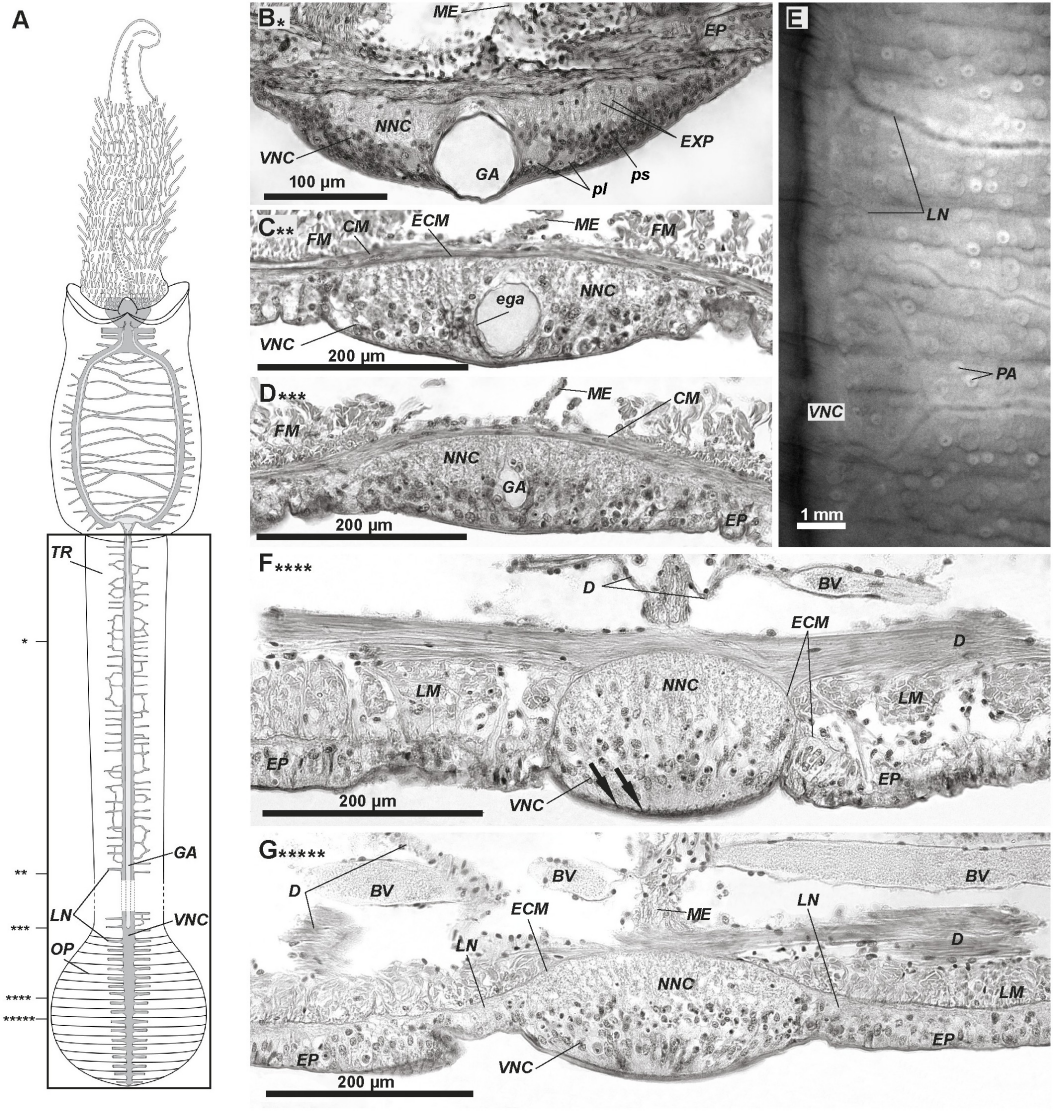
The ventral nerve cord in the trunk and opisthosome of *Riftia*. The trunk contains a single ventral nerve cord (*VNC*) (A) whose diameter gradually decreases towards the end of the trunk (B-D). A single giant axon extends to the border of the trunk and opisthosome (A-D). Numerous lateral neurite bundles (*LN*) arises from the VNC in the trunk, whereas in each segment of the opisthosome, only one pair of LN arises from the VNC (E-G). A–scheme of central nervous system: main elements in grey, giant axons in light grey. Anterior ends at top. Frame indicates area corresponding to histological sections (B-D, F, G) and light miscroscopical image (E). Stars show the levels of the corresponding sections. B–ventral nerve cord (VNC) structure in anterior trunk. C, D–VNC structure in mid- and posterior trunk, respectively; note reduction of giant axon diameter. E–lateral neurite bundles branching and anastomosing in trunk epidermis, anterior ends at top. F-G–VNC in mid- and posterior opisthosome. Arrows in (F): cuticular folds between cell borders. *ECM–*extracellular matrix, *BV–*blood vessels, *CM*–circular muscles, *D*–dissepiments, *ega–*cells coating *GA*, *EP*–epidermis, *EXP–*epidermal cell processes, *GA*–giant axons, *FM–*featherlike longitudinal muscles, *LG–*longitudinal lateral grooves, *LM–*longitudinal muscles, *LN–*circular neurite bundles, *lpnc–*lateral perikarya of *VNC*, *ME–*mesenterium, *NNC–*neuropile of VNC, *OP*–opisthosome, *PA*–cuticular plaque papillae, *pl*–large perikarya, *ps*–small perikarya, *TR*–trunk, *VNC–*ventral nerve cord.

**Fig 4:**
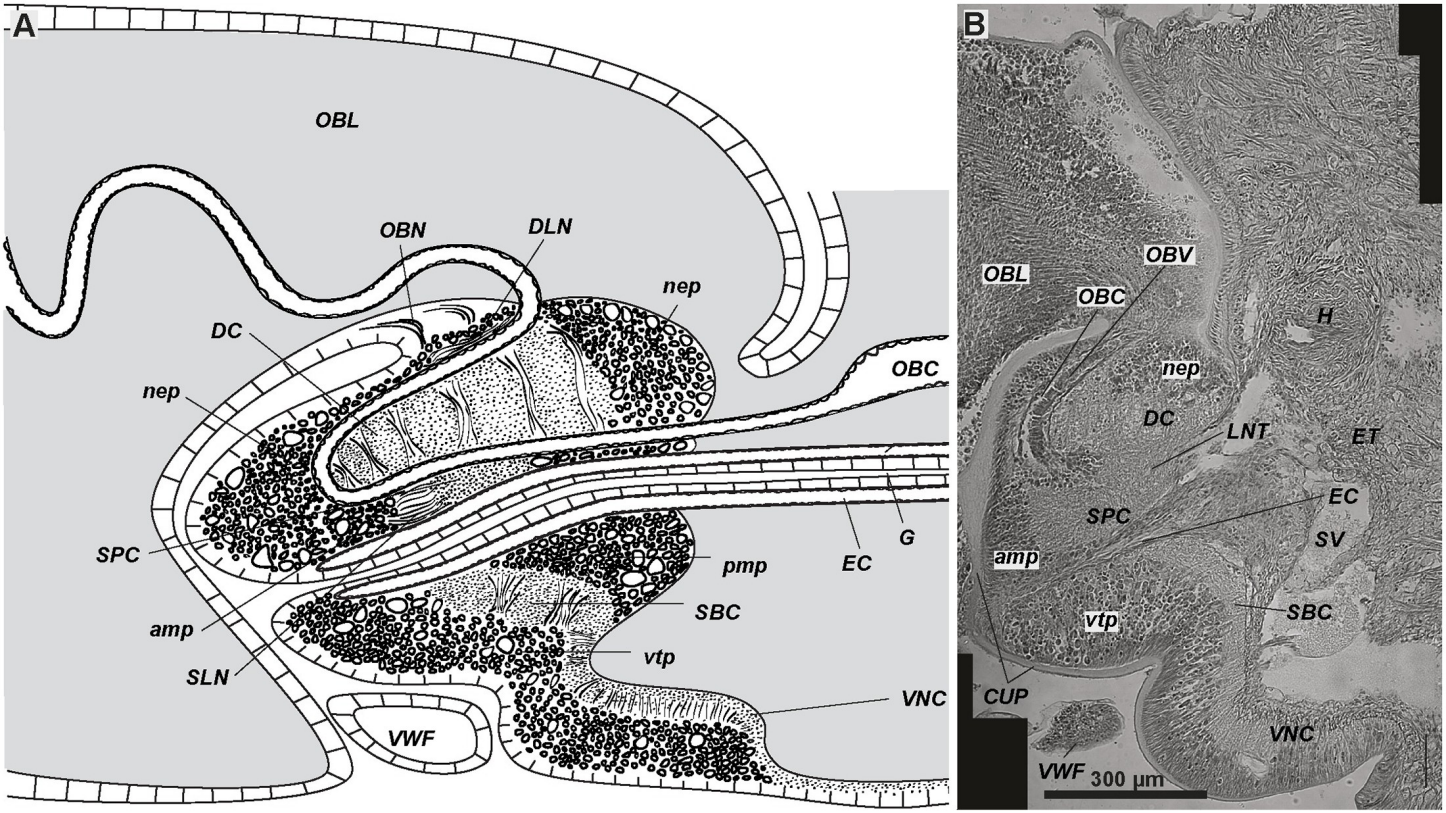
Brain of juvenile *Riftia* with a gut rudiment. Brain of *R*. *pachyptila* consists of dorsal and ventral parts divided by the enteral coelom (*EC*) containing the gut (*G*) (A, B). The dorsal part is associated with the supraesophageal ganglion, the ventral part with the subesophageal ganglion. A–idealised scheme of sagittal section of vestimentiferan brain, which consists of supraesophageal and subesophageal ganglia divided by the enteral coelom (EC). Anterior ends to left. B–one of the parasagittal sections of 8-mm-long juvenile; gut rudiment passes through brain. Anterior ends to left. *amp*–anterior median aggregation of perikarya, *CUP–*cuticle schield, *DC*–dorsal commissure, *DLN*–dorsal longitudinal bundles, *ET*–excretory tree, *G*–gut lumen, *EC*–enteral coelom, *H–*heart, *nep*–peripheral perikarya of lateral brain lobes, *OBC*–obturacular coelom, *OBL*–obturacular lobes, *OBN*–obturacular neurite bundles, *OBV*–obturacular blood vessels, *pmp*–posterior median perikarya aggregation, *SBC*–subenteral commissure, *SLN*–supraenteral longitudinal neurite bundles, *SPC*–supraenteral commissure, *SV*–sinus valvatus, *vtp*–tripartite ventral aggregation of perikarya, *VNC–*ventral nerve cord, *VWF–*collar of vestimental wings.

*Riftia*’s central nervous system is composed of a ventral **brain** and **ventral nerve cord** (*B*, *VNC*, Figs 1A, 2A, 3A and 4A).

**Fig 5:**
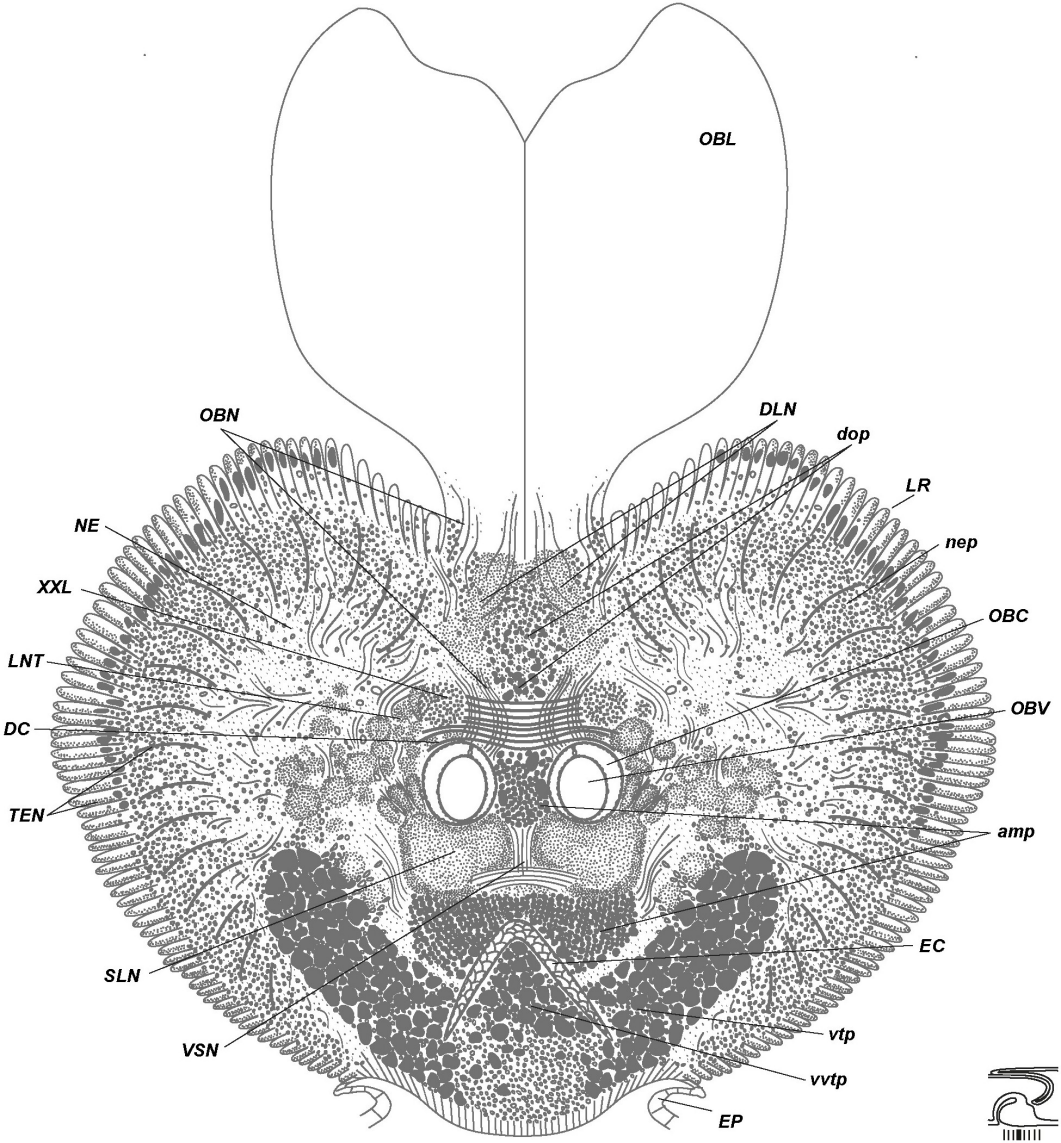
Anterior brain organization of *Riftia*. Scheme of histological cross section based on the anterior brain sections of a 79-mm-long specimen (S1 Fig). Level of section shown at diagram at bottom right. The enteral coelom (EC) enables demarcating position of supra- and subesophageal elements. Enteral coelom (*EC*) overlain by structures of supraesophageal ganglion: peripheral perikarya (*nep*) surround neuropile of lateral brain lobes (*NE*). Pair of *NE* connected *via* dorsal commissure (*DC*). Obturacular neurite bundles (*OBN*) enter *DC*. Structures of subesophageal ganglion (tripartite ventral aggregation of perikarya) underlie enteral coelom (*EC*). *amp*–anterior median aggregation of perikarya, *DC*–dorsal commissure, *DLN*–dorsal longitudinal bundles, *dop–*dorsal aggregation of perikarya, *EP*–epidermis, *EC*–enteral coelom, *LNT*–longitudinal nerve tracts projecting from *VNC* into brain, *LR*–undifferential tentacle lamellae, *NE*–neuropile of lateral brain lobes, *nep*–peripheral perikarya of lateral brain lobes, *OBC*–obturacular coelom, *OBL*–obturacular lobes, *OBN*–obturacular neurite bundles, *OBV*–obturacular blood vessels, *SLN*–supraenteral longitudinal neurite bundles, *TEN*–neurite bundles of tentacles (palps), *VSN*–vertical supraenteral neurite bundles, *vtp*—tripartite ventral aggregation of perikarya, *vvtp–*ventral perikarya of *vtp*, *XXL–*pair of prominent bundles of large longitudinal nerve tracts (part of *LNT*).

**Fig 6:**
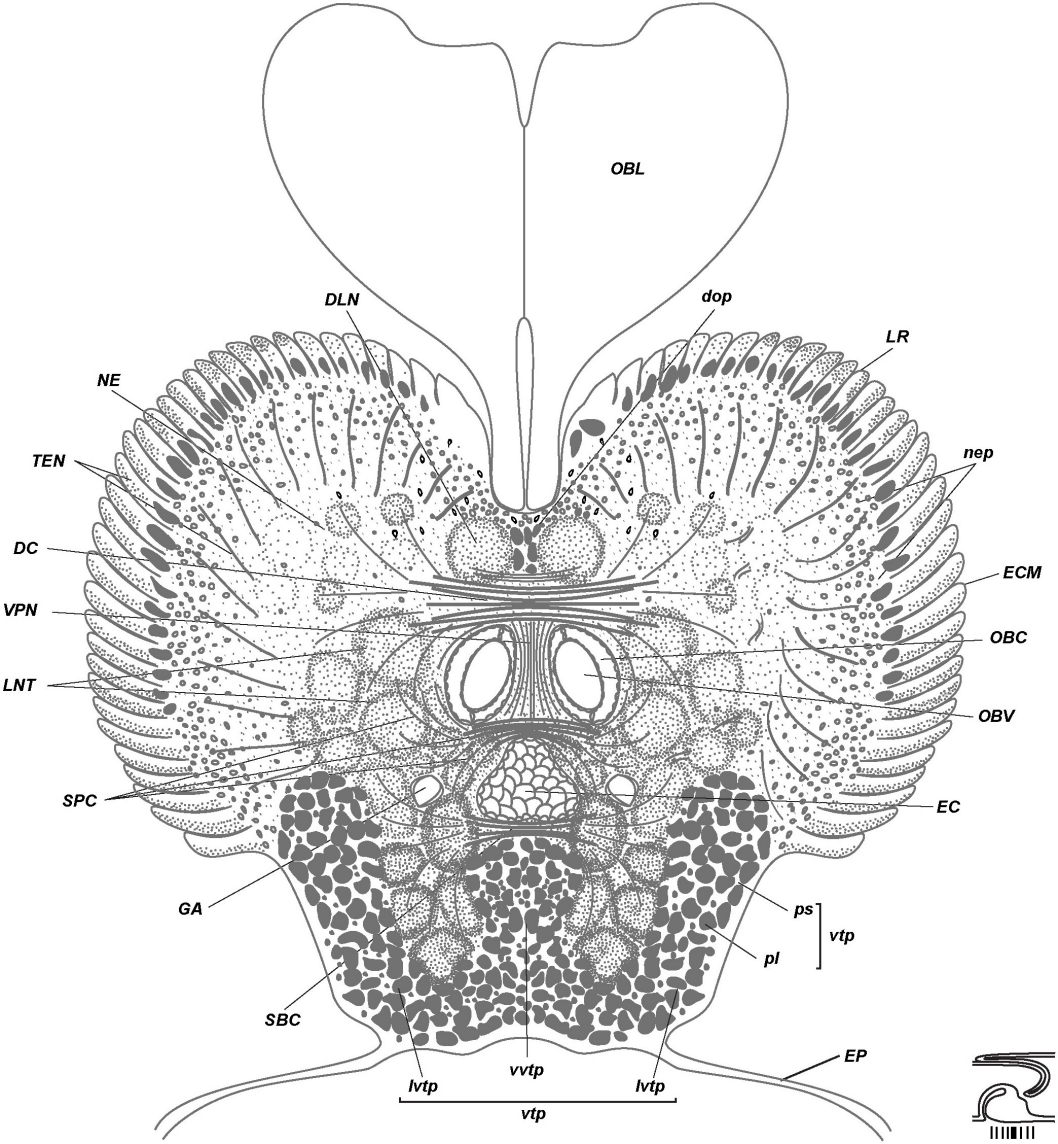
Middle brain organization of *Riftia*. Scheme of cross section based on the midbrain histological sections of a 79-mm-long specimen (S2 Fig). Level of section shown at diagram at bottom right. Midbrain’s elements of supraesophageal ganglion: neuropile of lateral brain lobes (NE) connected *via* dorsal commissure (*DC*) and supraenteral commissure (*SPC*). Midbrain’s elements of subesophageal ganglion: neuropile of tripartite ventral aggregation of perikarya (*vtp*) connected *via* subenteral commissure (*SBC)*. Longitudinal nerve tracts (*LNT*), as circumesophageal connectives, surround enteral coelom (*EC*). In dorsal brain part they give rise to DC, SPC, whereas in ventral brain they are connected *via* SBC. *DC*–dorsal commissure, *DLN*–dorsal longitudinal bundles, *dop–*dorsal aggregation of perikarya, *EP*–epidermis, *GA*–giant axons, *EC*–enteral coelom, *ECM–*extracellular matrix, *LNT*–longitudinal nerve tracts projecting from *VNC* into brain, *LR*–undifferential tentacle lamellae, *lvtp–*ventrolateral perikarya of *vtp*, *NE*–neuropile of lateral brain lobes, *nep*–peripheral perikarya of lateral brain lobes, *OBC*–obturacular coelom, *OBL*–obturacular lobes, *OBV*–obturacular blood vessels, *pl*–large perikarya, *ps*–small perikarya, *SBC*–subenteral commissure, *SPC*–supraenteral commissure, *TEN*–neurite bundles of tentacles (palps), *vtp*–tripartite ventral aggregation of perikarya, *VPN–*posterior vertical median bundles.

**Fig 7:**
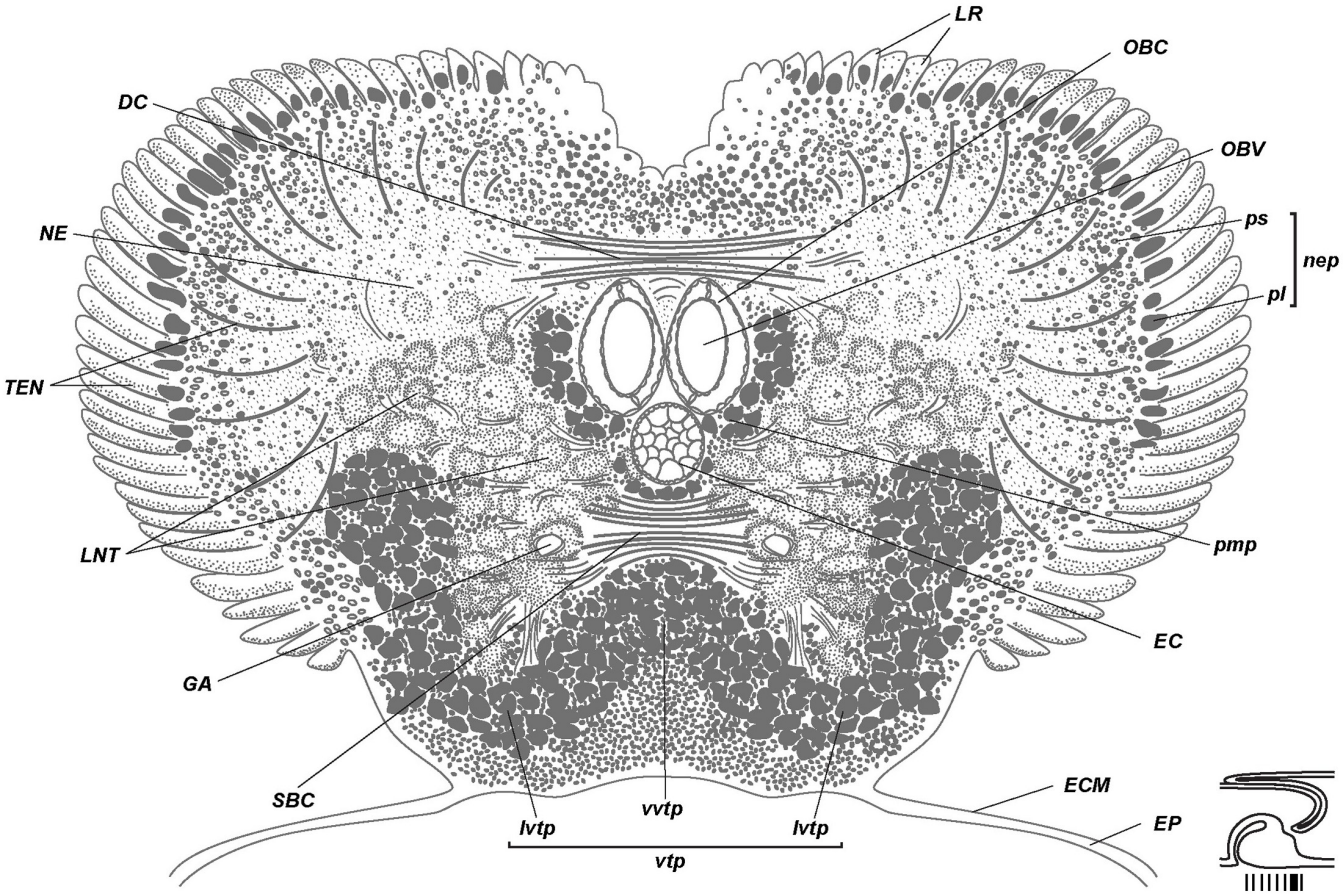
Posterior brain organization of *Riftia*. Schematic cross section based on the posterior brain histological sections of a 79-mm-long specimen (S3 Fig). Level of section shown at diagram at bottom right. Posterior brain elements of supraesophageal ganglion: neuropile of lateral brain lobes (*NE*) connected *via* dorsal commissure (*DC*). Posterior brain elements of subesophageal ganglion: neuropile of tripartite ventral aggregation of perikarya (*vtp*) connected *via* subenteral commissure (*SBC)*. Longitudinal nerve tracts (*LNT*) project from ventral nerve cord (*VNC*) into brain. *DC*–dorsal commissure, *ECM*–extracellular matrix, *EP*–epidermis, *GA*–giant axons, *EC*–enteral coelom, *LNT*–longitudinal nerve tracts projecting from ventral nerve cord into brain, *LR*–undifferential tentacle lamellae, *lvtp–*ventrolateral perikarya of *vtp*, *NE*–neuropile of lateral brain lobes, *nep*–peripheral perikarya of lateral brain lobes, *OBC*–obturacular coelom, *OBV*–obturacular blood vessels, *pl*–large perikarya, *pmp*–posterior median perikarya aggregation, *ps*–small perikarya, *SBC*–subenteral commissure, *TEN*–neurite bundles of tentacles (palps), *vtp*–tripartite ventral aggregation of perikarya, *vvtp–*ventral perikarya of *vtp*.

The ventral brain lies in the anteriormost vestimentum (Figs 1A, 4A and 4B). Two brain lobes form a heart-like structure in transverse sections (Figs 5–7, S1–S3 Figs). A dorsal furrow between the brain lobes encloses the bases of the obturacules (*OBL*, Figs 4A, 5 and 6, S1 Fig). Posteriorly, the excretory tree adjoins the brain (*ET*, Fig 4B). The whole brain lies inside the epithelium, and no basal laminae separate the brain from the epidermis (*EP*, Figs 4–7, S1–S4 Figs). A thick layer of cuticle (schield, or plate) protects the apical surface of the brain (*CUP*, Fig 4B, S1–S3 Figs). Furthermore, a collar of vestimental wings encloses the ventral brain (*VWF*, Figs 1B and 4). The brain of the 80-mm-long specimen is 1 mm high, 1 mm long, and 2 mm wide.

Tentacle lamellae originate at the brain periphery of the dorsal, lateral and ventrolateral sides of the brain (Figs 5–7, S1–S3A and S5 Figs). The posterior brain gives rise to lamellae only on the dorsal surface (Figs 6 and 7, S3A Fig), whereas on the anterior brain the tentacle lamellae occupy dorsal, lateral and ventrolateral surfaces (Fig 5, S1 and S2 Figs). Lamellae appear on the dorsal side of the brain. Here, they are the least differentiated (S5 Fig). The ventrolateral lamellae are bilayered (read below about the epidermis of the external and internal walls) and extend towards the anterior end of the tentacle crown. They remain undifferentiated over part of their length and then separate into individual tentacles. Thus, the undifferentiated tentacle lamellae (*LR***)** are lamellae that are not divided into separate tentacles (*LR*, Figs 5–7, S1–S3A and S5 Figs).

Three coelomic channels pass through the brain tissue: one pair of **obturacular coeloms** with blood vessels and an unpaired **enteral coelom** (*OBC*, *EC*, Figs 4–7, S6 Fig). In juvenile individuals the enteral coelom comprises the **gut rudiment** (*G*, Fig 4A). In larger specimens the enteral coelom is occupied by mesenchymal cells (Fig 6). In the anterior brain the enteral coelom has a «Λ» shape when viewed in the transverse profile (Fig 5). The oral siphon is preserved in juvenile *Riftia* (34 mm long), and in 79-mm-long individuals the intestine rudiment remains in the coelomic channel running through the brain. In larger individuals, only the coelomic channel remains.

The ventral nerve cord (*VNC*) connects to the brain *via* **longitudinal nerve tracts** (*LNT*, Fig 1B–1E). Anteriorly to the **ventral ciliary field** (*CF*) the VNC splits into a **pair of strands** (*PNC*) connected to each other with transverse neurite bundles (Fig 2A–2D). The strands surround the ventral ciliary field (Fig 2B and 2C) and fuse into a single VNC at the border of the vestimentum and trunk before they extend along ventral midline to the end of the body (Fig 3A). The width of the prominent VNC can reach up to 1 mm in an 808-mm-long specimen (Fig 3B). Notably, its width decreases towards the posterior trunk (Fig 3C and 3D).

The VNC lies inside the epidermis (Fig 1E). The epidermal cells have a wide apical part underlying the cuticle and a basal process running towards the ECM (Fig 1C).

Apically, a thick cuticular layer (CU) protects the VNC, especially in the anteriormost part (Fig 1C). In the opisthosome the cuticle protecting the VNC makes folds between the apical parts of epidermal cells (arrows, Fig 3F).

### Dorsal brain structures

**Fig 8:**
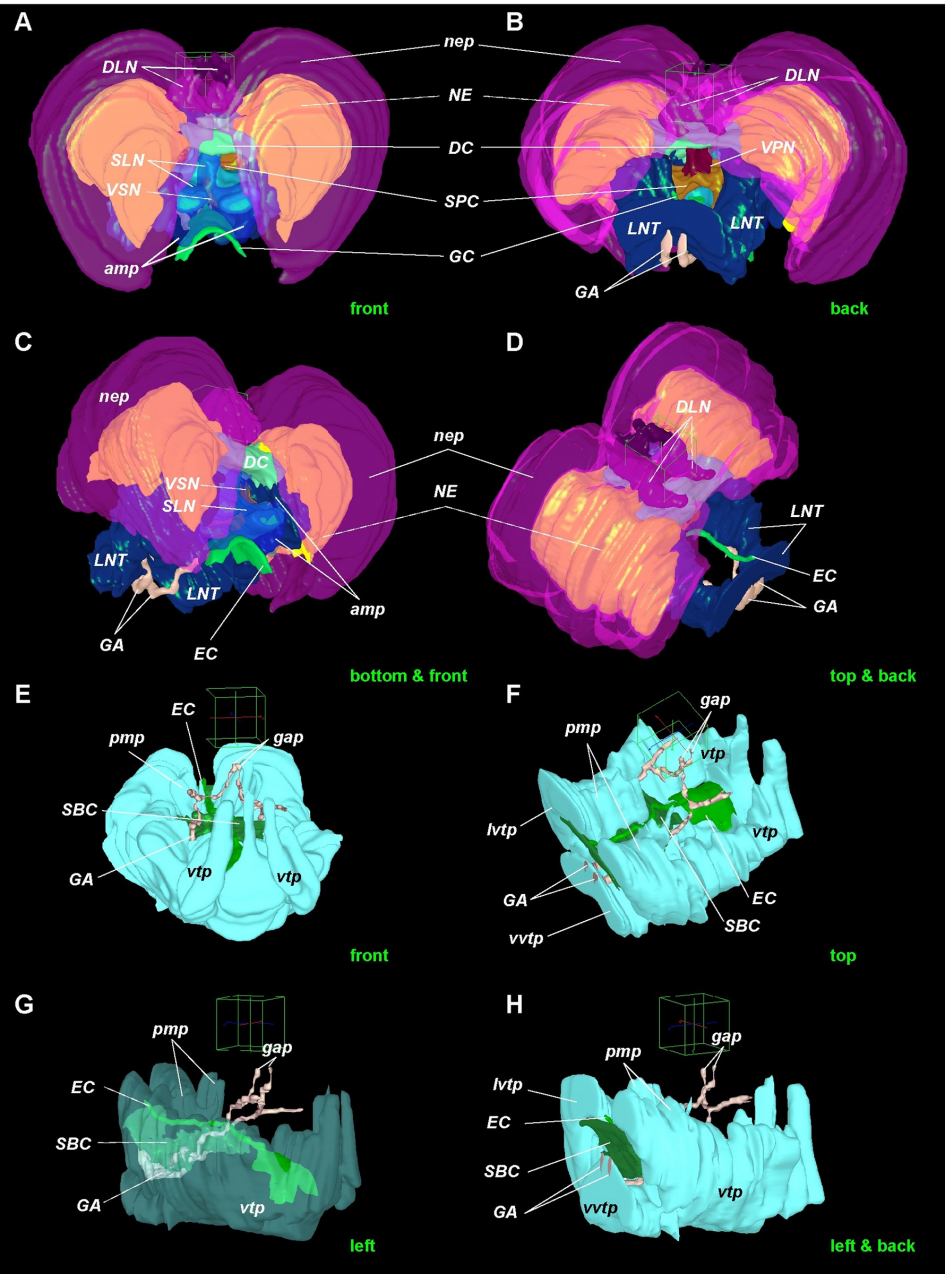
Supra- and subesophageal ganglia in *Riftia*. 3D models of the *Riftia* brain. Enteral coelom (EC) containing the gut demarcates the dorsal and ventral parts of the brain. Dorsal part homologous to supraesophageal ganglion, ventral part to subesophageal ganglion. A-D–supraesophageal neuronal elements. E-H–subesophageal neuronal elements in *Riftia* brain. View sides shown at bottom right of each image. Cube side 255 μm. Dashed lines: neural elements under transparent structures. *amp*–anterior median aggregation of perikarya, *DC*–dorsal commissure, *DLN*–dorsal longitudinal bundles, *GA*–giant axons, *gap–*giant perikarya, *EC*–enteral coelom, *LNT*–longitudinal nerve tracts projecting from ventral nerve cord into brain, *lvtp–*ventrolateral perikarya of *vtp*, *NE*–neuropile of lateral brain lobes, *nep*–peripheral perikarya of lateral brain lobes, *pmp*–posterior median perikarya aggregation, *SBC*–subenteral commissure, *SPC*–supraenteral commissure, *SLN*–supraenteral longitudinal neurite bundles, *VPN–*posterior vertical median bundles, *VSN–*vertical supraenteral neurite bundles, *vtp*–tripartite ventral aggregation of perikarya, *vvtp–*ventral perikarya of *vtp*.

**Fig 9:**
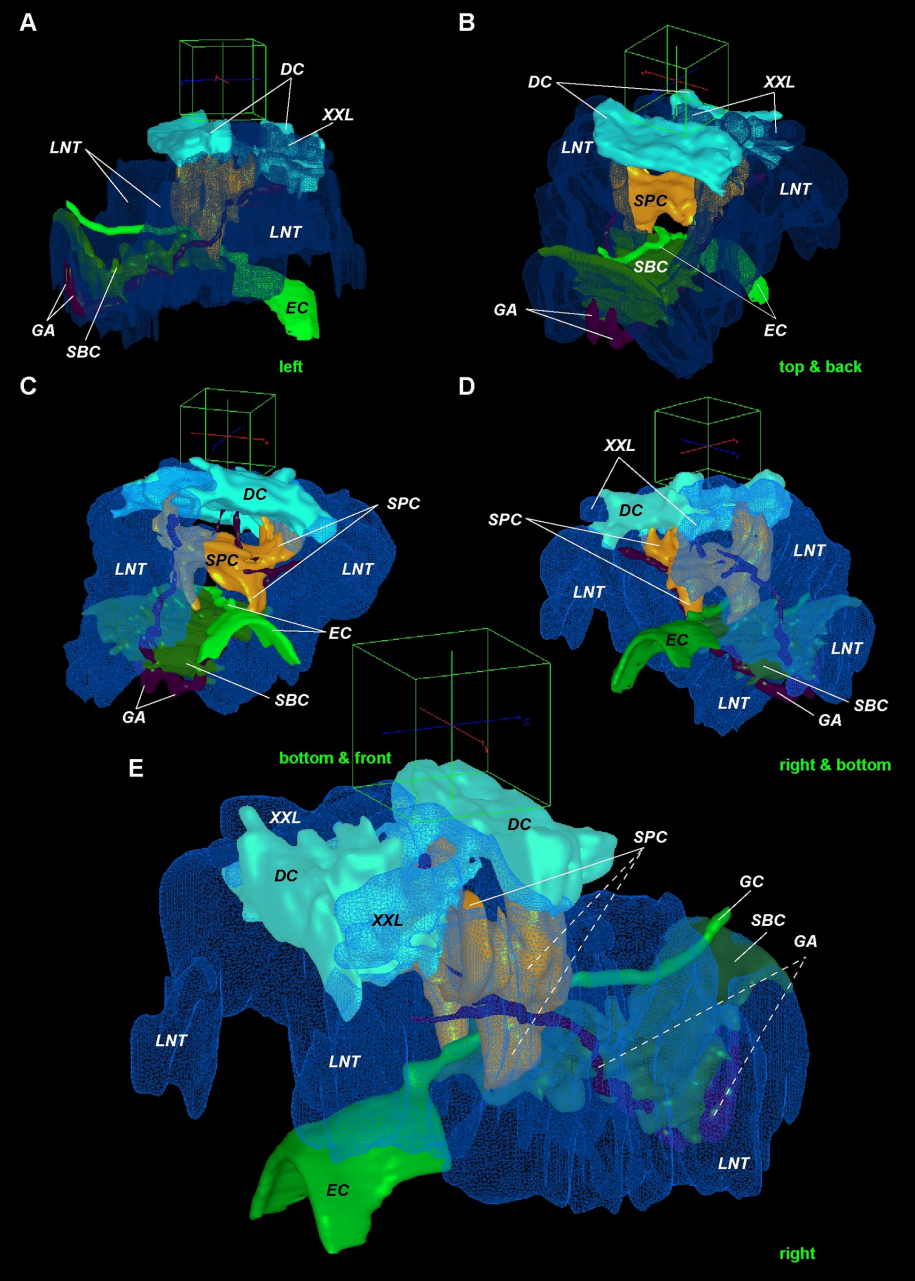
Longitudinal nerve tracts and main commissures in *Riftia* brain. 3D models of the *Riftia* brain. Longitudinal nerve tracts (*LNT*), as circumesophageal connectives, connect supra- and subesophageal ganglia and give rise to dorsal commissure (*DC*) and supraenteral commissure (*SPC*) in supraesophageal ganglion, and to subenteral commissure (SBC) in subesophageal ganglion. Giant axons running from giant perikaya lie in anterior DC (not shown) to ventral nerve cord. A-E–main commissures (dorsal, *DC*, supra-, *SPC*, and subesophageal, *SBC*) and longitudinal nerve tracts (*LNT*). The latter is homologous to circumesophageal connectives in other annelid brains. View sides shown at bottom right of each image. Cube side 255 μm. Dashed lines: neural elements under transparent structures. *DC*–dorsal commissure, *GA*–giant axons, *EC*–enteral coelom, *LNT*–longitudinal nerve tracts projecting from ventral nerve cord into brain, *SBC*–subenteral commissure, *SPC*–supraenteral commissure, *XXL–*pair of prominent bundles of large longitudinal nerve tracts (part of *LNT*).

The brain of *Riftia pachyptila* consists of the dorsal and ventral parts divided by the enteral coelom (Figs 4–8). The pair of prominent **longitudinal nerve tracts** (*LNT*) comes from the ventral nerve cord and connects the ventral and dorsal parts of the brain (Figs 1B–1D, 4B, 5–7 and 8A–8D). In the dorsal brain part the longitudinal nerve tracts have dorsal and ventral roots that are interconnected *via* **dorsal** (*DC*) and **supraenteral commissures** (*SPC)*, respectively (Figs 5, 6 and 9, S1–S3 Figs). In the ventral brain part the longitudinal nerve tracts are connected *via* the **subenteral commissure** (*SBC*, Figs 6, 7 and 9, S3 Fig).

Most of the dorsal brain is represented by a large paired **neuropile of the lateral brain lobes** (***NE***, Figs 5–8, S1–S3 and S7A–S7C' Figs). Nerve tracts of *NE* have various diameters, including a **pair of large bundles** of thick fibers, each fibre being 6–11 μm in diameter (*XXL*, Figs 5, 9A, 9B, 9E and 10A–10F, S2B, S7D–S7H, S9B and S9C Figs).

**Fig 10:**
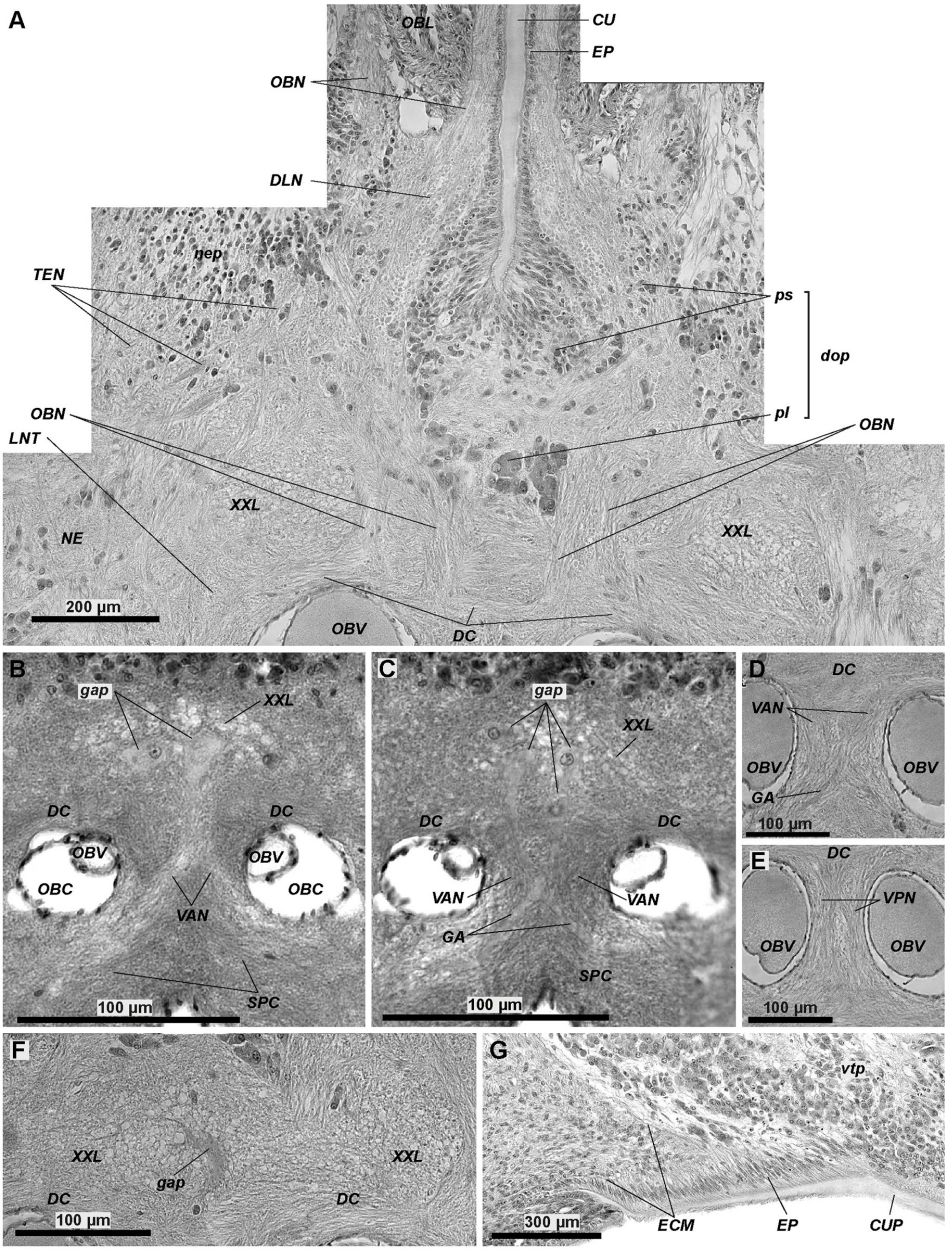
Histological details in the anterior brain of *Riftia*. In the anterior brain, the dorsal commissure gives rise to the obturacular neurite bundles (*OBN*) (A). Giant axons (*GA*) extend from giant perikarya (*gap*) and follow the anterior vertical median bundles (*VAN*), which cross each other (B-D). Anterior and posterior vertical median bundles (*VAN*, *VPN*) are present. The latter do not cross each other (D, E). Thick cuticle layer (cuticular shield, *CUP*) protects apical surface of brain (G). A–obturacular neurite bundles (*OBN*) connecting with dorsal commissure (*DC*). B, C–giant perikarya with clear nuclei in juvenile brain. D–anterior vertical median bundles (*VAN*) composed of giant axons (*GA*). E–posterior vertical median bundles (*VPN*) with no giant axon. F–giant perikarion degrading in brain of 79-mm-long male. *G*–cuticular plate protecting brain (*CUP*). *ECM*–extracellular matrix, *CU–*cuticle, *CUP–*cuticle schield, *DC*–dorsal commissure, *DLN*–dorsal longitudinal bundles, *dop–*dorsal aggregation of perikarya, *EP*–epidermis, *GA*–giant axons, *gap–*giant perikarya, *LNT*–longitudinal nerve tracts projecting from *VNC* into brain, *NE*–neuropile of lateral brain lobes, *nep*–peripheral perikarya of lateral brain lobes, *OBC*–obturacular coelom, *OBL*–obturacular lobes, *OBN*–obturacular neurite bundles, *OBV*–obturacular blood vessels, *pl*–large perikarya, *ps*–small perikarya, *SPC*–supraenteral commissure, *TEN*–neurite bundles of tentacles (palps), *vtp*–tripartite ventral aggregation of perikarya, *VPN–*posterior vertical median bundles, *VAN–*anterior vertical median bundles, *XXL–*pair of prominent bundles of large longitudinal nerve tracts (part of *LNT*).

Numerous **tentacle neurite bundles** (***TEN***) extend radially from the *NE* to the bases of the tentacles (Figs 5 and 6, S1–S3 and S5 Figs). The latter are arranged into rows with the fused bases, so-called tentacle lamellae. Each tentacle lamella represents a thin fold of the epidermis and closely adjoins the next lamella (S5A–S5D Fig). The **epidermis of the external lamellae wall** (***OEP***) is thin, whereas the **epidermis of the internal wall** (***IEP****)* is thicker and contains the basiepithelial *TEN* (S5A Fig).

The neurites of tentacles (*TEN*) originate from the neuropile of the lateral brain lobes (*NE*), which in turn come from the pair of roots of the prominent **longitudinal nerve tracts (*LNT***, Figs 5 and 6; S1, S7 and S9 Figs). *LNT* bifurcate into two roots, ventral and dorsal ones, each of which is interconnected *via* dorsal and supraenteral commissures. Neurites of *TEN* mostly originate from the dorsal root of the *LNT*, then the ventral root (S9 Fig). *TEN* does not come form the commissures of LNT (either from the dorsal or supraenteral ones).

In the anterior dorsal brain, the *LNT* are connected to each other by thick **dorsal commissures** (*DC*, Figs 5, 6, 9 and 10A, S1–S3A and S7D–S7H Figs). The latters run over the paired obturacular coelomic channels and adjacent to their anterior loops (S8F and S8G Fig). The *DC* is divided into two almost equal parts: the anterior and posterior commissures (Fig 9A, 9B and 9E, S8F and S8G Fig). Both anterior and posterior *DC* are structured in the dorso-ventral direction and comprise several layers of neurite bundles which are visible in transverse section (up to 5 levels in the 79-mm-long specimen, S2A Fig). Thus, up to 9–11 ventro-dorsal vertical bundles run through the *DC*, clearly visible in sagittal and parasagittal sections (Fig 4A).

Two pairs of **obturacular neurite bundles** (*OBN*) extend from the dorsalmost area of the brain from the anterior dorsal commissure to the bases of the obturacular lobes (Figs 5 and 10A, S1 and S9A–S9F Figs). Each pair of obturacular bundles (left and right) gives rise to neurite bundles in the epidermis of the inner and outer sides of the obturacular lobes. In that area, neurite bundles run vertically, whereas in the dorsal part of the obturacules they change orientation and run in posterior-anterior direction.

In the midbrain the longitudinal nerve tracts (*LNT*) are connected to each other *via* a **supraenteral commissure** (*SPC*) running over the enteral coelomic channel and under the obturacular channels (Figs 4A, 9B–9D, 10B and 10C, S3A, S8D, S8E, S9J and S10 Figs). Together, the *SPC* and *DC* give rise to two pairs of prominent **supraenteral longitudinal neurite bundles** (*SLN*), which run forward to the anteriormost brain (Fig 8E, 8F and 8G). Notably, the *SLN* extending from the DC start as a single bundle, then ramify into two bundles termed **vertical supraenteral neurite bundles** (*VSN*, Fig 5, S2B and S8A–S8C Figs). Both pairs of *SLN* represent stalk-like neuropiles, which are connected to an **anterior median aggregation of perikarya** (*amp*; more below; Figs 4 and 5, S2 and S8 Figs).

The dorsalmost side of the midbrain (close to ECM layer) bears a pair of **dorsal longitudinal bundles** (*DLN*, Figs 4A, 5, 6, 8A–8D and 10A, S1–S3A and S9A–S9C Figs). They start from a **dorsal aggregation of perikarya** (*dop*) in the midrain (Figs 5 and 10A, S1 and S2 Figs) and run along the dorsal groove of the brain.

Short **anterior vertical median bundles** (*VAN*) pass between the obturacular coeloms in the midbrain (Fig 10B–10D, S10 Fig). They run in the ventro-dorsal direction and connect the dorsal and supraenteral commissures. The neurites of the anterior vertical median bundles cross: for example, the neurites from the right root of the supraenteral commissure extend to the left roots of the dorsal commissure (Fig 10D, S10B Fig).

**Posterior vertical median bundles** (*VPN*, Figs 6, 8B and 10E, S3A and S10 Figs) are located posterior to the anterior vertical median bundles. They do not contain any crossing bundles and connect the supraenteral commissure and the posterior dorsal commissure.

**Peripheral perikarya of the lateral brain lobes** (*nep*, Figs 4–7 and 8A–8D, S1–S3, S5, S6, S7I and S7J Figs) are represented by two layers: inner layer of small perikarya, 5 μm (*ps*), and outer layers of big ones (*pl*), 20 μm (Fig 7, S3A and S5A Figs). In juvenile specimens with fewer tentacle lamellae, the small perikarya are grouped into distinct lobules which correspond to the tentacle lamellae. In bigger specimens with more tentacle lamellae, the arrangement of small perikarya is more regular. Accordingly, the *nep* does not represent a true cluster or aggregation, but rather the layer of the somata spreading over the dorsal and lateral surface of the brain. In the anterior part of the brain the peripheral zone of perikarya expands significantly and covers laterally a **tripartite ventral aggregation of perikarya** (*vtp*, Figs 5–7, S1–S3 and S6–S8 Figs, more about *vtp* read below).

The dorsal groove of the anterior brain bears a **dorsal aggregation of perikarya** (*dop*) which lies in the inner sides of the obturacules entering the brain (Figs 5, 6 and 10A, S1–S3A Figs). It contains two layers of perikarya: in contrast to the peripheral perikarya, there are inner big perikarya and outer small ones (Figs 5 and 10A).

The pair of **anterior median aggregations of perikarya** (*amp*) is the most anterior symmetrical accumulation of small somata (Figs 4, 5, 8A and 8C, S1, S2 and S8 Figs). It dorsally adjoins the enteral coelomic channel. These aggregations consist of hundreds of small somata whose nuclei occupy almost the whole cytoplasm. The somata are arranged in rows of 8–15 cells (S1 Fig). A pair of thick supraenteral longitudinal neurite bundles (*SLN*) form stalk-like neuropiles connected to the small somata of the *amp*.

### Ventral brain structures

Most of the neuropile of the ventral brain is occupied by paired prominent **longitudinal nerve tracts** (*LNT*, Figs 1B–1D, 6, 7, 8B–8D, 9 and 10A, S1–S3, S7 and S9B–S9J Figs), which are continuations of nerve fibers from the ventral nerve cord (Fig 1B–1D). As the longitudinal nerve tracts enter the brain, each of them runs along the lateral sides of the obturacle and enteral coelomic channels and gradually rises to the anterior dorsal side of the brain (Figs 5–7).

In the ventral brain the pair of longitudinal nerve tracts is connected under the enteral coelomic channel *via* the **subenteral commissure** (*SBC*, Figs 4, 6, 7, 8E–8H and 9, S3 and S8D–S8G Figs), which is a continuation of the transverse neurites in the ventral nerve cord (*CNC*, Fig 2B). The ventralmost part of the brain, situated under the enteral coelom, is occupied by the **tripartite ventral aggregation of perikarya** (*vtp*, Figs 4–7, 8E–8H and 10G, S1–S3, S6, S7A–S7C, S7I, S7J, S8D and S8E Figs) comprising small and big perikarya (Fig 6). In transverse sections it is divided into three lobes: one ventral and two ventrolateral ones (*vvtp*, *lvtp*, Fig 7, S2B, S3 and S6 Figs). In the posterior brain, the distinct lobes are more prominent than in the anterior brain (Figs 6 and 7, S3 Fig). In the anterior brain the unpaired ventral lobe (*vvtp*) adjoins the ventral side of the enteral coelomic channel (Figs 5, 8F and 8G, S1, S2 and S6D–S6F Figs), whereas in the posterior brain it adjoins the subenteral commissure (Figs 6 and 7, S3 Fig). In the posterior brain the ventrolateral perikarya of the tripartite aggregation receive about 9–12 branching neurite bundles parted off the *LNT (*S3 Fig*)*. In the posterior brain, two groups of big perikarya–**posterior median perikarya aggregations** (*pmp*)–extend from the tripartite aggregation and run along the left and right sides of three coelomic channels (Figs 7 and 8E–8H, S3B and S6F Figs).

### Giant perikarya and axons in the brain

In adult specimens, **the giant axons** are present in the midbrain originating from at least two pairs of **the giant perikarya** (*GA*, *gap*, Figs 6, 7, 8E–8H and 9, S3, S9G–S9L and S10B–S10G Figs). One pair starts in the dorsal commissure, runs inside the crossing anterior vertical median bundles (*VAN)* between the obturacle coeloms (Fig 10D and 10F, S9G–S9L and S10E–S10G Figs). Another pair starts in the longitudinal nerve tracts, on the lateral sides of the obturacle coeloms (S9G–S9L Fig). Both pairs of the giant axons join ventrally to the obturacle coeloms (S9G–S9L Fig), their cytoplasms fuse, and they continue their paths along the longitudinal nerve cord and enter the ventral nerve cord as a single pair (Fig 1A and 1B).

Notably, in adult specimens, the dorsal commissure has only one pair of the giant perikarya, whose nuclei and nucleoli are degraded (Fig 10F), whereas the juveniles have two pairs of the dorsal giant perikarya (Fig 10B and 10C). Considering the number of the giant perikarya in juveniles and that the giant perikarya in adult animals degrade, we assume at least 3 pairs of giant perikarya for the vestimentiferans.

In the posterior brain the giant axons extend to the ventral nerve cord (Fig 9, S9G–S9J Fig). Transversally the giant axon represents the 20–25 μm round profile with light cytoplasm and enveloped by flattened cells with dark nuclei (S3 Fig). We were unable to distinguish between several neurite bundles within the giant fibers.

### Ventral nerve cord

Within the vestimentum, the neuropile of the paired ventral nerve cord (VNC) consists of two lateral longitudinal nerves (*LNT*, Fig 1B–1D) connected *via* transverse (commissural) neurite bundles (*CNC*, Figs 1B, 1D and 2B). A pair of giant axons lies in the central part of the VNC (Fig 1A–1D). Numerous small perikarya form two lateral and one central accumulation (*lpnc*, cpnc, Fig 1C and 1D) which are continuations of the ventral tripartite aggregation of the ventral brain (*vvtp*, Fig 1B and 1C).

Around the ventral ciliary field, each strand of *PNC* contains the epidermal cells, basal neuropile, apical perikarya, and a single fiber representing the giant axon envelopped by the coating cells (*ega*) (Fig 2D). Most perikarya lie externally to the giant axon in each strand. The ciliary field consists of columnar ciliary epidermal cells (Fig 2C). Their basal parts contain commissural neurite bundles (*CNC*) which form a net-like structure and connect the strands with each other (Fig 2A, 2B and 2D).

In the trunk the VNC has an invariable diameter–the neuropile has no swellings and is separated by the giant axon into two longitudunal strands (Fig 3A–3D). The epidermal cell processes extend to the ECM inside the neuropile (*EXP*, Fig 3B). The VNC perikarya spread along the left and right sides of the giant axon (Fig 3B–3D). Both small (3.5 μm) and big (20 μm) perikarya (*ps*, *pl*, Fig 3B) are present. The giant axon extends to the border of the trunk and opisthosome (Fig 3D).

In the opisthosome, the arragement of the apical somata and the basal neuropile of the VNC is comparable to the rest of the body (Fig 3F and 3G). There is no giant axon; all perikarya are small.

### Segmental nerve bundles

In the anteriormost vestimentum, several thick transverse **lateral neurite bundles** part off the ventral nerve cord (*LN*, Fig 1A). Three pairs were present in a 16-mm-long specimen. The first, most prominent pair extends to the anterior collar and forms **neurites of** the **vestimental wings** (*VWN*, Fig 1A and 1B). At the level of the ciliary field, many irregular bundles branch off the lateral neuropile of the VNC strands and extend into the epidermis of the vestimental wings (Fig 2A and 2E). Transverse neurite bundles branch off from the single VNC in the trunk. They ramify and anastomose (Fig 3E). In a 79-mm-long specimen, lateral bundles part off the cord, yielding 350–360 pairs of such bundles in the trunk. In each opisthosomal segment, a pair of lateral bundles leaves the neuropile of the VNC (Fig 3A, compare F&G).

## Discussion

### Ventral nerve cord in vestimentifera

All described species of vestimentiferans have a uniform structure of the ventral nerve cord (*VNC*), with variations evident only in the length of giant axons and the organization of perikarya aggregations within the trunk [16,18,19,22,23,26,40]. The VNC in *Ridgeia piscesae* and *Lamellibrachia satsuma* consist of a central neuropile and two lateral rows of perikarya, thus showing a paired structure [18,22], whereas *Riftia* (present study) and *Oasisia alvinae* exhibit single layers of apical perikarya and a basal neuropile [23]. Furthermore, a median groove runs along the midline of the VNC in the opisthosome of *O*. *alvinae* [23].

A pair of giant axons extends from the pair of giant perikarya reported in the vestimentiferans *Ridgeia*, *Riftia*, *Oasisia*, *and Lamellibrachia* [17,22–24]. Notably, the giant axons terminate at different levels within the trunk VNC: in *L*. *luymesi*, giant axons terminate in the anterior part of the trunk [16], in *L*. *barhami* the latter extend somewhat further back [41], and in *R*. *piscesae*, *O*. *alvinae* and *Riftia* they extend up to the border between trunk and the first opistosome segment [22,23]. Previous studies reported that a pair of giant perikarya is retained in juvenile *R*. *piscesae* and *O*. *alvinae* in the mid-dorsal part of the brain [22–24,26]. In the present analysis, we found two pairs of the giant neurons in the dorsal commissure of juvenile *Riftia* (Fig 10B and 10C). Moreover, the lateral branches of giant axons (S10E–S10G Fig) indicate the possible presence in earlier stages of at least one more pair of giant perikarya in the lateral areas of the neuropile. Thus, each giant fiber in *Riftia* is a product of the fusion of at least three pairs of axons.

### Ventral nerve cord in siboglinidae

Siboglinids have an intraepidermal ventral nerve cord (*VNC*) with evenly dispersed perikarya [18,21,23,27,28,30,31,34–36]. The authors of earlier studies failed to note the paired structure of the VNC, the association with the ciliary field, and the giant axons.

All siboglinids have a paired VNC. First, the VNC is paired in the anterior segment of the vestimentiferans (the vestimentum), frenulates (the forepart) and *Osedax priapus* (the trunk), which were suggested to be homologous to each other [35]. Second, vestimentiferans and large frenulates have a pair of giant axons (absent in *Osedax* and *Sclerolinum)*. Third, in *Sclerolinum*, with the single nerve cord, the VNC bifurcates into two strands around the ventral ciliated field. Fourth, *Osedax* species (females and males) exhibit a distinct pair of widely separated strands of the VNC in the trunk [21,27,28,30,34–36].

The ventral ciliary field, a structure conserved in most siboglinids, lies in the anterior body part: in the trunk of frenulates, in the vestimentum of vestimentiferans, within the forepart of *Sclerolinum* and within the anterior trunk of female *O*. *priapus* [21,30,35,36]. Although the ciliary field in frenulates and in both the vestimentiferans and *Sclerolinum* lies in different regions, it always originates from the larval neurothroch [42,43]. In the developing larvae of the frenulate *Siboglinum fiordicum*, the anterior part of the neurotroch extends to the future forepart, whereas the posterior part of the neurotroch extends to the future trunk. In developing *S*. *fiordicum*, only the posterior part of the neurotroch remains in the trunk of adults [42,43]. In adult vestimentiferans the ciliary field (= the anterior part of the neurotroch) remains in the vestimentum [21,38]. We assume that in adult frenulates and vestimentiferans, different parts of the neurotroch remain, possibly due to different larval life modes. Vestimentiferan larvae swim for a long time in the water, whereas in frenulates they settle and simultaneously undergo metamorphosis. Among the questions for future studies are the functions of the ciliary fields in siboglinids as well as whether the lateral ciliary bands of *Osedax* females originate from the neurotroch or not.

Perikarya do not form accumulations along most of the length of the VNC, i.e. in forepart/vestimentum and trunk, but their number increases in the region of annular chaetae, as reported in the frenulate *Lamellisabella zachsi* [27,28]. In short opisthosomal segments of the frenulate *Siboglinum fiordicum* the perikarya possibly form the ganglionic masses [29,30]. In contrast to the vestimentiferans’ anchoring opisthosome, the frenulate opisthosome is designed to protrude out of the posterior tube opening and to dig into the sediment [30]. Due to this high mobility, the VNC in the frenulate opisthosome forms three strands with paired somata masses in each segment in *Siboglinum fiordicum* [29,44].

Giant axons in the vestimentiferans *Ridgeia*, *Riftia and Oasisia* [22–24] were found to extend up to the posterior end of the trunk. In large frenulates such as *Spirobrachia* and *Lamelisabella*, a pair of giant axons extend from the giant unipolar perikarya located in the brain [28,30]. In small frenulates such as *Nereilinum* there is only one giant axon, and it runs only along one side of the ventral ciliary field [30]. In frenulates, the giant axons extend only until the girdle of hook-shaped chaetae located approximately in the mid-trunk, whereas in the vestimentiferans the latter structure is detectable until the end of the trunk. The giant axons support a rapid contraction of the longitudinal musculature, serving the so-called "flight response"–in the frenulates and vestimentiferans it is the retraction of the body deep into the tube when threatened (i.e. by claws of crabs). To do so, frenulates are anchored to the wall of the tube with their girdle chaetae, whereas the vestimentiferans use chaetae of the opisthosome for this purpose. That may explain why the giant axons extend only to the girdle in the frenulates, and to the opisthosome in the vestimentiferans. Interestingly, *Osedax* and *Sclerolinum* lack giant axons.

Thus, the VNC in siboglinids is arranged in a very similar way. In the anterior part of the body the paired strands are associated with the ventral ciliary field. In all groups, the VNC lies entirely within the epidermis and contains the giant axons. The VNC is not ganglionated over most of its length. The question for future studies is whether the frenulates and *Sclerolinum* have a ganglionized VNC (includingthe opisthosome).

### Ventral nerve cord in siboglinids and annelids

The siboglinids have an intraepidermal paired medullary ventral nerve cord (VNC) containing giant axons (except *Sclerolinum* and *Osedax*) and associated with the ventral ciliary field. The intraepidermal position of the VNC is also known in species of Opheliidae, Spionidae, Syllidae, Maldaniidae, Cossuridae, Polygordiidae and Protodrillidae as well as in the basally branching Chaetopteridae, Magelonidae and Oweniidae [45–48]. Siboglinids are one more annelid group with the intraepidermal VNC, additionally underlining that such a condition might be part of the annelid ground pattern.

Based on the presence of the paired nerve cords in the hypothetical sister clades Cirratulida, Sabellida and Spionida [47,49–53], and on the paired organization of the nerve cord in anterior segments in siboglinids, we conclude that this configuration of the VNC (at least in the anterior segments) is an ancestral feature for siboglinids (Fig 12). This pattern of the VNC are found in the larval stages of Errantia, Sedentaria and basally branching clades [54–62]. Although in adult annelids VNC is organized in surprising range of levels–paired, trineural, or pentaneural [45,47,63,64]–the paired state is suggested as being ancestral for annelids [65].

Most annelids exhibit a uniformly structured VNC along the worm body as either medullar or ganglionated [45,47,63,64]. In case of siboglinids, it is medullar in *Osedax* [35], *Riftia* (here), and most of the length of the VNC in frenulates [29,30].

The arrangement of the segmental neurite bundles in vestimentiferans is similar to that in oweniids [61,66,67]: numerous and anastomosing neurites in the long anterior segments, and condensed single bundles in the short posterior segments. This convergent pattern may reflect segment elongation.

Giant axons and giant perikarya are common among annelids, especially in large forms [64,68]. A common feature of most annelids is the multicellular or unicellular giant fibres extending from the giant somata. The latter are usually situated in subesophageal ganglia and/ or other segmental ganglia. In vestimentiferans, one pair of giant perikarya (as opposed to the at least three pairs we detected in *Riftia*) are located within the supraesophageal ganglion. Among annelids, only in sabellids such as large *Myxicola infundibulum* and *Sabella pavonina*, and spionids, such as *Prionospio steenstru*, do the giant perikarya lie in the supraesophageal ganglion. In the others they lie in the VNC [68].

Vestimentiferans together with most remaining siboglinids have the ventral ciliary field bordered by a pair of VNC strands. The ciliary field is not common in sexually mature annelids. It is known in presumably progenetic *Dinophilus gyrociliatus* [69,70], where it is used for gliding. Note that the tiny frenulate *Nereilinum murmanicum* uses this ciliary field to glide vertically along the tube as well [31]. Other functions of this structure in siboglinids remain theoretical [17].

### Brain organization in vestimentiferans

The differences in the brain structure of vestimentiferan species lie mainly inbrain shape and the presence/absence of cuticular structures [16,18,19,22,23,26,40].

*Riftia pachyptila*'s brain is heart-shaped in transverse section, with significantly developed dorso-lateral lobes (Figs 5 and 8A–8D). In *Ridgeia piscesae* it is triangular in transverse section and has a wide ventral side [22]. In contrast, this organ is oval-transverse in *Lamellibrachia luymesi* [16]. The two latter species have less developed dorso-lateral lobes than *Riftia* (S5 Fig). *Rifia* possesses 340 tentacles per lamellae and 335 lamellae on each side of the obturaculum, whereas 70 lamellae in *Escarpia* seem to be the maximum value in other vestimentiferans [19,71,72]. This helps explain the enlarged dorso-lateral lobes in *Riftia’*s brain. Importantly, despite the brain shape differences, tentacle nerves originate from the same dorso-lateral areas of the brain neuropile in *Riftia* and all other vestimentiferans.

Finally, a prominent cuticle shield protects the ventral side of the brain. This shield has direct contact with the tube or ambient environment in all studied vestimentiferans (Fig 10G) [16,22,23,26]. The dorsal and frontal sides of the brain are covered by tentacles and obturacules (Figs 4 and 5). Moreover, the brain can be penetrated by cuticle rods and plates extending from the cuticle of tentacle lamellae, as observed in *L*. *luymesi*, *R*. *piscesae*, *O*. *alvinae*, but not in *Riftia* [16,23,26].

### Brain anatomy of vestimentiferans and annelids

The vestimentiferan brain lies completely within the epidermis at the anteriormost part of the vestimentum. It comprises the large and dense mass of the neuropile and perikarya, giving the impression of a single entity. The “brain” was once suggested to be a product of the fusion of supra- and subesophageal ganglia [20]. The juvenile vestimentiferans exhibit the gut rudiment, which can be used to help homologize the brain parts of the gutless siboglinids with the relevant parts of the annelid brain (supraesophageal ganglion) and postoral segmental paired ganglion of the ventral nerve cord (subesophageal ganglion). Below we provide detailed homologization.

**Fig 11:**
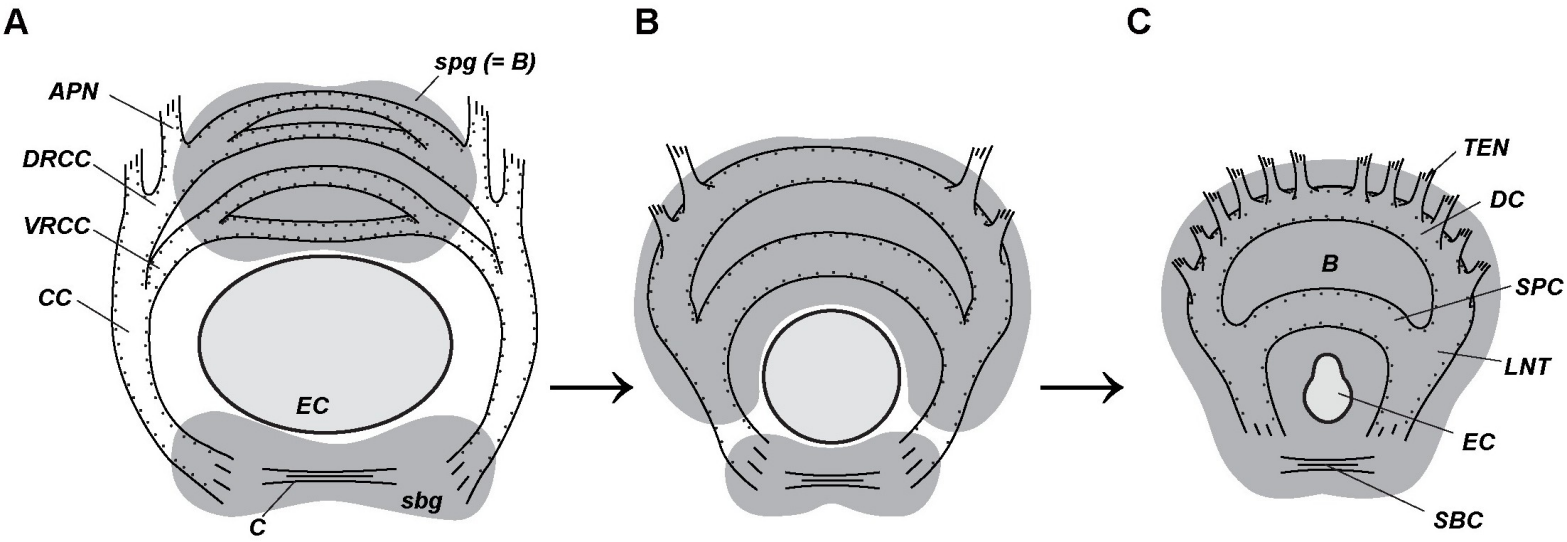
Hypothetical vestimentiferan brain origin. Orrhage and Müller [47] showed the general presence of 4 transverse commissures in the brain (a.k.a. supraesophageal ganglion) of numerous annelids (A), which join through their roots and circumesophageal connectives to the ventral nerve cords. The palp neurites extend from the dorsal root of the circumesophageal connectives in annelids. In vestimentiferans (C), the “brain” is a fusion of the supra- and subesophageal ganglia. Longitudinal nerve tracts (*LNT*), as circumesophageal connectives, connect the ganglia. The dorsal (*DC*) and supraenteral (*SPC*) commissures connect transversely the paired structures of the supraesophageal ganglion, whereas the subenteral commissure (*SBC*) connects transversely the structures of the subesophageal ganglion. The innervation of tentacles from the DC makes them homologous to peristomial palps of other annelids. Hypothetical ancestral state of vestimentiferan brain (B) is transitional between annelid brain (A) and vestimentiferan brain (C). Dorsal side to the top. A–supra- and subesophageal ganglia in annelids (after [47]). B–hypothetical transitional state. C–vestimentiferan brain. *APN–*neurite bundles of palps, *B*–brain, *C*–commissure of *sbg*, *CC–*circumesophageal connectives, *DC*–dorsal commissure, *DRCC–*dorsal (posterior) root of the *CC*, *EC*–enteral coelom, *LNT*–longitudinal nerve tracts projecting from ventral nerve cord into brain, *sbg*–subesophageal ganglion, *SBC*–subenteral commissure, *spg*–supraesophageal ganglion (the brain in annelids), *SPC*–supraenteral commissure, *VRCC*–ventral (anterior) root of *CC*, *TEN*–neurite bundles of tentacles (palps).

Longitudinal nerve tracts (*LNT*) can be homologized with the circumesophageal connectives of annelids (Figs 9 and 11). First, like annelid circumesophageal connectives [45,73], the *LNT* are somata-free nerve bundles interconnecting the postoral segmental paired ganglia of the ventral nerve cord (*VNC*) and the supraesophageal ganglion in the anteriormost part of the brain in *Riftia* (Figs 5–7; S1–S3 Figs). Second, like annelid circumesophageal connectives [47,49,74–76], the *LNT* run ventrally to the the enteral coelomic channel within the *VNC* and the posterior brain, extend from the *VNC* anteriorly, run along the lateral sides of the gut, and rise to the dorsal “brain” in *Riftia*. Third, like annelid circumesophageal connectives, the *LNT* give rise to two pairs of roots: dorsal and ventral ones (Fig 6; S3A Fig). The dorsal roots are interconnected *via* the dorsal commissures, the ventral roots *via* the supraenteral commissure (Fig 9). Fourth, in annelids, the lateral and ventral sides of the circumesophageal connectives and the ventral nerve cord are associated with ganglia cells of the postoral ganglia of the *VNC* [49]. In *Riftia*, both the *LNT* and *VNC* are surrounded laterally and ventrally by the ventral tripartite aggregation of the perikarya (*vtp*).

The part of the vestimentiferan “brain” lying dorsally to the enteral coelomic channel can be homologized with the supraesophageal ganglion (Figs 4, 8A–8D and 11), the part of the brain ventral to the enteral coelomic channel with the subesophageal ganglion (Figs 4, 8E–8H and 11).

The supraesophageal ganglion in *Riftia*, like in other annelids (and certain other taxa), is the most prominent anterior condensation of neurons [73]. This is the first reason. Second, in *Riftia* this ganglion has the peripheral perikarya (*nep*) surrounding the neuropile of the lateral brain lobes (*NE*) (Fig 5; S1 Fig). Third, like in various other annelids [47], in the dorsal part of the brain (which we homologize with the supraesophpageal ganglion) we found two of the most prominent commissures (dorsal and supraenteral) connected *via* dorsal and ventral roots to the circumesophageal connectives (= longitudinal nerve tracts, *LNT*). These commissures are also present in the vestimentiferans *Oasisia* and *Ridgeia* (Fig 7 in [22], Fig 4c in [23]). Fourth, in annelids, the somata of the brain may be arranged in distinct groups around the central neuropile, with a few somata usually located ventrally [45]. In *Riftia*’s supraesophageal ganglion, most somata groups are located dorsally and laterally; the ventral side is almost free of somata, except for the anterior median aggregation of perikaria (*amp*, Figs 5–7; S1–S3 Figs).

The subesophageal ganglion of annelids and arthropods is the first paired ventral ganglia (or postoral segmental paired ganglion) interconnected *via* circumesophageal connectives with the supraesophageal ganglion [45,47,49,63,64]. In *Riftia*, this is the prominent tripartite aggregation of big perikarya (*vtp*) on the ventral side of its “brain”. The *vtp* lies behind the reduced mouth siphon (Fig 4A and 4B) and ventrally to the enteral coelomic channel containing the gut (Fig 8E–8H). As in annelids, the *vtp* (= subesophageal ganglion) ventrally and laterally surrounds both the *LNT* (= circumesophageal connectives) and *VNC*.

Commissural cell clusters at the junction of the dorsal and ventral roots of the circumoesophageal connectives are widespread among “polychaetes” (including most sedentarian species) [47,49] and are suggested to be a ground pattern character of annelids [64]. In the present analysis, we hypothesize that the lateroventral perikarya of the tripartite ventral aggregation (*lvtp*), which are situated in the posterior brain in front of the junction of the dorsal and supraenteral commissures into the *LNT* (= circumesophageal connectives), are the commissural cluster of somata (Fig 7; S3 Fig). Here, some neurite bundles branch off and extend from the *LNT* to the small perikarya of the *lvtp*. Moreover, in annelids, the commissural cell cluster very often fuses with the first ventral ganglion [49,64]. In *Riftia*, as well as in *Oasisia* and *Ridgeia* [22,23], the *lvtp* is always associated with the *vtp*, which is the first ventral ganglion.

Mushroom bodies, or the “corpora pedunculata” known in arthropods, annelids and possibly in some flatworms and molluscs, are the stalk-like neuropil forming several lobes and surrounded by a cap of small neuronal somata termed globuli cells [45,63,64,77–82]. The globuli cells possess a minute amount of cytoplasm and are especially rich in chromatin. They form columns comprising from ten to thousands of cells [49,63,64,73]. Accordingly, the mushroom-like structures in *Riftia* could be a complex formed by the anterior pair of the median aggregation of perikarya (*amp*) and two pairs of supraenteral longitudinal neurite bundles (*SLN*). The *SLN* could be homologous to a stalk-like neuropile of the linear neurite bundles. This is surrounded distally by *amp*, which could be homologous to the small globuli cells arranged in rows (Fig 5; S1 and S2 Figs). The whole complex of *amp* and *SLN* adjoins the supraesophageal ganglion. In the vestimentiferan *Oasisia*, the *amp* and *SLN* were also detected as *P-medN* and *vCS*, respectively (Fig 5a in [23]). Mushroom bodies are found in many errant and in some sedentary annelids such as the serpulid *Serpula*, the alvinellid *Paralvinella hessleri*, and sabellids, including *Chone* and *Euchone* species [49,64,80,83]. As in *P*. *hessleri* [83] and *E*. *papillosa* [49], the mushroom-like structures in *Riftia* do not occupy the typical dorso-lateral position in the brain, but instead extend anteriorly. Interestingly, the chemosynthetic *P*. *hessleri* exhibits theses structures, which potentially play a specific chemosensory role in the vent environment [83].

Most annelid palps (in 22 out of the 32 polychaete families) are innervated from the roots of the circumesophageal connectives; single palps are innervated from the dorsal commissures (see Fig 2 in [47]) [49]. The innervation of the numerous tentacles of *R*. *pachyptila* is provided by the tentacle neurite bundles (*TEN*) radially emanating from the neuropiles of the lateral brain lobes (*NE*), which in turn receive the neurites from both roots of the longitudinal nerve tracts, dorsal and supraenteral (*LNT*, Figs 7, 8A–8D and 11C). The latter are homologous to the dorsal and ventral roots of circumesophageal connectives. The palps are the only appendages of annelids to receive the neurites from both the ventral and dorsal roots. The conclusion is that the tentacles of *Riftia* are innervated from both roots of the circumesophageal connectives as are most annelid palps. Why do vestimentiferans have palps but no other annelid anterior appendages or organs? Various antennae in annelids are innervated only from certain parts of the dorsal root of connectives [47]. The stomatogastric neurites in annelids extend from the commissures and not from connective roots [47]. No oral filaments, buccal appendages, nuchal organ or eyes have been detected in adult vestimentiferans.

Previously, vestimentiferan tentacles were homologised with polychaete palps [11]. Nonetheless, this homology was considered doubtful based on differences in the external and internal structures (lack of ciliated grooves, absence of longitudinal support rods and the presence of the afferent and efferent blood vessels inside each tentacle) [38]. Our data on the innervation of *Riftia* tentacles proves the annelid palp hypothesis of vestimentiferan tentacles (Fig 11). In vestimentiferans (especially *Riftia*), parts of the longitudinal nerve tracts and neuropile of the lateral brain lobes are incomparably larger than the corresponding neural structures in annelids. This is because the tentacle apparatus of vestimentiferans is highly developed. A similar correlation between the sizes of tentacle crowns and brains is clearly evident in oweniids and sabellids. In oweniids, featuring simple gill tentacles and pigmented eyes, the brain is a simple transverse commissure followed by the layer of perikarya passing in the epidermis dorsal to the digestive tract [66,67]. In contrast, in sabellids–featuring a large tentacle crown serving for food collection, pigmented eyes and statocysts–the brain consists of four main transverse commissures, dorsal and ventral roots of the circumesophageal connectives, an additional four commissures, anterior loops of the dorsal root, blood vessel neurites, giant perikarya and axons, nuchal nerves, neurites of statocysts and eyes, palp nerves, two pairs of mushroom bodies, as well as lateral and median clusters of brain [49].

We agree with Jones and Gardiner [20] that the brain is a result of the union of the supra- and subesophageal ganglia. For the first time, in the dorsal part of the vestimentiferan brain (= supraesophageal ganglion), we found homologues of the dorsal and ventral pairs of the transverse commissures, mushroom bodies, commissural cell clusters, palp neurite bundles, and giant somata. Furthemore, we found homologues of circumesophageal connectives and the subesophageal ganglion. We have found possible homologs that were considered to be absent. Thus, we do not confirm the hypothesis on the simplicity of the vestimentiferan brains [18].

Our comparative anatomical approach shows that the structure of the vestimentiferan brain and VNC does not go beyond the diversity of neuronal structures in Annelida. Within Sedentaria, vestimentiferans have complex brains, which are comparable to brains of their close sister-clades, Cirratuliformia and Spionida/Sabellida, as well as to representatives of possible basally branching sedentarians, like Orbiniidae [47]. Whereas siboglinid brains (with several transverse commissures and palp neurites) do not resemble brains of more distant sedentarian clades like terrebellids and pectinariids [13]. Brains of the latters comprise a single transverse commissure that gives rise to the neurites of buccal appendages and to the stomatogastric neurites [47].

### The homologous structures in siboglinid CNS

The vestimentiferan genera *Riftia*, *Ridgeia*, *Oasisia*, *Lamellibrachia* and bone-eating worm *Osedax* are the only siboglinids whose brains have been studied in detail [17,18,21,23,26,34,35,84, 85].

**Fig 12:**
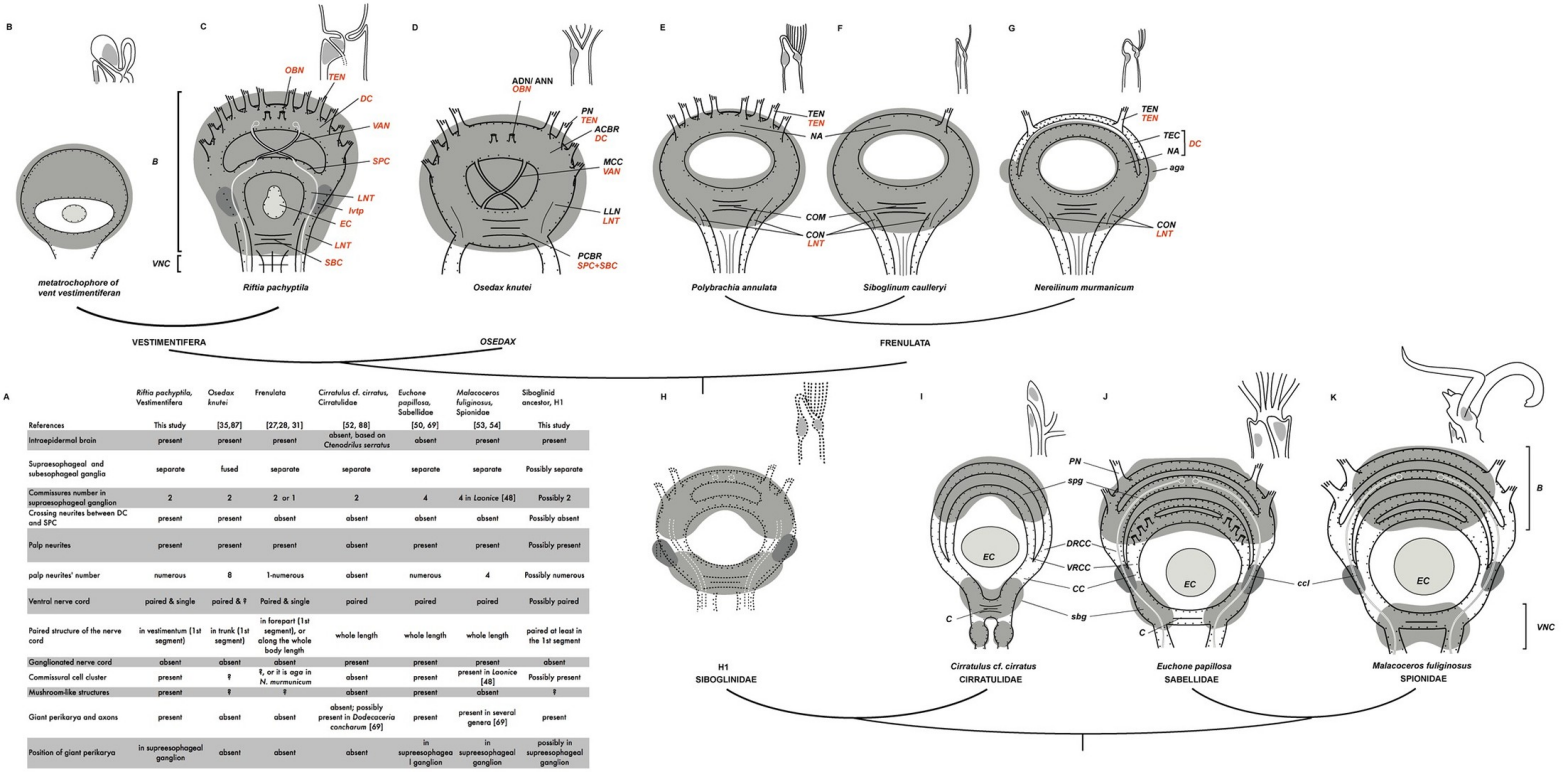
Reconstruction of hypothesized neural ancestor traits of the siboglinid central nervous system. Main neural characters of siboglinids and annelid sister groups used to reconstruct neural archetype of siboglinid ancestor. For neural characters to homologize CNS in siboglinids and annelid sister groups, see table (A) and diagrams (B-K). In table (A), “?” means “not known because not studied in detail”. We suggest the neural archetype of the siboglinid ancestor to have had separate supraesophageal (= brain) and subesophageal ganglia, two commissures of circumesophageal connectives which were interconnected by at least one pair of crossing neurites and possibly gave rise to numerous neurites into palps. Giant perikarya are places in the supraesophageal ganglion. Our hypothesis: the VNC of the siboglinid ancestor was paired (or at least paired in the anterior part) and non-ganglionated, with a pair of giant axons. Relation among siboglinid groups based on a combination of recent phylogenetic trees: frenulate clade resolved based on cladistic analysis [7], interrelationship of siboglinid clades on phylogenetic and phylogenomic data [6,85, 86, 87], annelid outgroups on phylogenomic data [13]. Neural diagrams include left (upper row) and dorsal views (lower row) of the anteriormost body. Anterior/ dorsalside are at top. Perikarya position shown in grey. Cerebral elements drawn based on larvae of vestimentiferan *Riftia pachyptila* [25] (B); adult *Riftia pachyptila* (this study) (C); *Osedax knutei* (= *O*. “nudepalp E” [35,85]) (D); frenulates *Polybrachia annulata* (E) *Siboglinum caulleryi* [27,28] (F) and *Nereilinum murmanicum* [31] (G), cirratulid *Cirratulus cf*. *cirratus* [51,68,88] (I), sabellid *Euchone papillosa* [49,68] (J), and spionid *Malacoceros fuliginosus* [47,50,52,53,64,68] (K). Reconstructed archetype H1 has a combination of the hypothesized ancestral siboglinid states (H). Dashed lines: hypothesized neuronal elements in siboglinid ancestor. *ACBR*–anterior commissure of brain, *ADN*–antero-dorsal nerve, *aga*–ganglion-like aggregation at base of *TEC*, *ANN*–anterior nerve net, *B*–brain, *C*–commissure in *sbg*, *CC–*circumesophageal connectives, *ccl*–commissural cell cluster; *COM*–commissure, *CON*–connective, *DC*–dorsal commissure, *DRCC–*dorsal (posterior) root of the CC, *EC*–enteral coelom, *LLN*–lateral longitudinal neurite bundles in brain, *LNT*–longitudinal nerve tracts projecting from *VNC* into brain, *MCC*–middle cross commissure, *NA*–nerve ring (after [28]) or brain ring (after [31]), *OBN*–obturacular neurites, *PCBR*–posterior commissure of brain, *PN*–palp nerve, *TEC*–tentacular commissure, *TEN*–neurite bundles of tentacles (palps), *SBC*–subenteral commissure, *sbg*–subesophageal ganglion, *SPC*–supraenteral commissure, *spg*–supraesophageal ganglion, *VAN–*anterior vertical median bundles, *VNC–*ventral nerve cord, VRCC–ventral (anterior) root of CC.

We suggest a homologization of the dorsal commissure of *Riftia* (*DC*) and anterior commissure in *Osedax* (*ACBR*, [35]). They are situated in the anteriormost parts of the brains and also give rise to the neurite bundles of the homologous anterior structures (Fig 12). First, palps are innervated by the palp neurites branching off the roots of the circumesophageal connectives in both *Riftia* (*TEN*) and *Osedax* (*PN*). Second, the obturacule neurites in vestimentiferans (*OBN*) and the antero-dorsal nerves or the anterior nerve net in *Osedax* (*ADN* and *ANN)* give rise from the middle parts of the dorsal commissures (Fig 12; see Fig 2 in [35]). A future task will be to trace the vertical bundles branching from the dorsal commissure in other siboglinid species as well as to find homologous neuronal structures among siboglinids and annelids.

We suggest that the anterior and posterior bundles of the posterior commissure in *Osedax (PCBR)* are homologous to the bundles of the supraenteral and subenteral commissures in *Riftia (SPC*, *SBC*, Fig 12). *Osedax* lacks a gut, we use the remaining intracerebral neural structures for this homologization. First, crossing neurites connect the dorsal commissure to anterior bundles of the *PCBR* in *Osedax* (*MCC*, [35]) and to the *SPC* in *Riftia* (*VAN*). Second, the *VNC* is interconnected by the posterior bundles of the *PCBR* in *Osedax* and the *SBC* in *Riftia*.

We consider the lateral longitudinal bundles in *Osedax* (*LLN*) and the longitudinal nerve tracks in *Riftia* (*LNT*) to be homologous to each other and to the annelid circumesophageal connectives (Fig 12). Both the *LLN* in *Osedax* and the *LNT* in *Riftia* are connected by the main transverse commissures, their roots give rise palp neurites, and they are continuations of the VNC.

The brain organization in frenulates, sister group to the remaining siboglinids [2,86], is important in analyising the ancestral state of the siboglinid brain, but remains to be studied in detail. Notably, studied frenulates have commissures in the epidermis of the dorsal body side: a single dorsal commissure in *Polybrachia annulata*, *Siboglinum caulleryi* [27,28], and two dorsal commissures in *Nereilinum murmanicum*, *S*. *modestum* and *S*. *subligatum* [31]. These commissures in frenulates give rise the neurite bundles to anterior tentacles, which we preliminarily homologise with the annelid palps based on the origin from the dorsal root of the circumesophageal connectives (Fig 12).

The *Sclerolinum* brain is a very simple structure lying entirely in the ventral epidermis and having two layers: apical perikarya and basal neuropile [36]. We currently lack sufficient details to homologize it with other siboglinids and annelids.

## Conclusions

A comparative neuroanatomical analysis of the siboglinids and the annelid sister clades enables us to hypothesize that the last common ancestor of siboglinids had separate supra- and subesophageal ganglia, two roots of the circumesophageal connectives giving rise to neurite bundles to numerous palps, a commissural cell cluster, a paired ventral nerve cord, and had giant perikarya in the supraesophageal ganglion with paired giant axons running within the paired nerve cord (Fig 12). The strands of the VNC and the giant axons probably fused posteriorly. Notably, within Sedentaria siboglinids form the sister clade with the Cirratuliformia, and Spionida/Sabellida having the complex brain with the similar structures, like several transverse commissures and palp neurites. Siboglinids do not exhibit reduction in neuroanatomical complexity, like terebellids and pectinariids, which have no traces of commissures of the circumesophageal roots as well as no palp neurites. Future neuroanatomical studies should reveal if within the Sedentaria the simplification of the brain was the one of the trends of their evolution.

Our study provides definitive closure in the dispute on the origin of the siboglinid tentacles and proves them to be palps based on their innervation, as originally proposed by Orrhage [47]. This conclusion was supported by tracing the direction of evolution of palp nerve roots within Annelida in light of current phylogenetic analyses [13,87]. The palps and their nerve roots are a recognised ground pattern of the annelids [47,64,88,89]. Indeed, palp-bearing species are present in all three major groups. Therefore, we assume that palp-bearing species have palp neurites. Within the basally branching groups and Errantia, each clade comprises palp-bearing species, including the Amphinomidae, Chaetopteridae, Magelonidae [47,90], Eunicida, Phyllodocidae, and Protodriliformia [91–96]. Within Sedentaria, only the basally branching lineages have palps, such as the Orbiniida (Nerellidae; in Orbinidae the palp nerves are present [95,97]), Spionida/ Sabellida [47,49,64], and Siboglinidae/ Cirratuliformia (within the Cirratuliformia: Flabelligeridae and Acrocirridae [47]. More distant sedentarian clades are palp-less, e.g. Clitellata, Terebelliformia/ Arenicolidae, and Opheliidae/ Capitellidae/ Echiura (*Ophelia* has palp nerve roots though). The sedentarian groups are distinguished by a high variety in the number of palp nerve roots: from zero to 335 pairs; whereas this variety is lower in Errantia and basally branching groups. There are 0, or 3–4 pairs of palp roots within basally branching species versus 1–6 within the errantian species [64]. Accordingly, sedentarian species exhibit the greatest variety in the number of palp nerve roots. Their number changed from several to many palps (as in vestimentiferans) or was reduced completely (as in clitellates). Future research should examine if and how the heterogeneity of palp nerve roots arrangements in annelids is based on their functionality.

## Supporting information

S1 Fig Anteriormost brain of *Riftia*. Anteriormost brain contains elements of supraesophageal ganglion (above enteral coelom, *EC*) and subesophageal ganglia (under *EC*). In supraesophageal ganglion, the dorsal commissure (*DC*) connects the longitudinal nerve tracts (LNT) projecting from ventral nerve cord into brain. Obturacular neurite bundles (*OBN*) enter *DC*. As a part of the subesophageal ganglion, the tripartite ventral aggregation of perikarya (*vtp*) expands to anterior part of brain. Level of section shown in diagram, right lower corner. Section level is between sections shown in S2A and S2B Fig. *amp*–anterior median aggregation of perikarya, *CU–*cuticle, *CUP–*cuticle shield, *DC*–dorsal commissure, *DLN*–dorsal longitudinal bundles, *dop–*dorsal aggregation of perikarya, *EP*–epidermis, *EC*–enteral coelom, *LNT*–longitudinal nerve tracts projecting from VNC into brain, *LR*–undifferentiated tentacle lamellae, *NE*–neuropile of lateral brain lobes, *nep*–peripheral perikarya of lateral brain lobes, *OBC*–obturacular coelom, *OBL*–obturacular lobes, *OBN*–obturacular neurite bundles, *OBV*–obturacular blood vessels, *SLN*–supraenteral longitudinal neurite bundles, *TE*–free tentacles, *TEN*–neurite bundles of tentacles (palps), *vtp*–tripartite ventral aggregation of perikarya.
(TIF)

S2 Fig Anterior and middle brain organization of *Riftia*. Comparison of anterior (A) and middle (B) brain sections. Anterior brain (A) contains anterior median aggregation of perikarya (*amp*) and huge areas of neuropile of lateral brain lobes (*NE*). NE occupies most of dorsal and lateral sides of brain and gives rise to the neurite bundles of tentacles (palps). Huge volume of *NE* reflects very high number of tentacles (palps) in vestimentiferans, especially in *Riftia* (up to 335 lamellae pairs, *LR*). In posterior brain (B), ventral and ventro-lateral sides are occupied by tripartite ventral aggregation of perikarya (vtp) comprising ventral (*vvtp*) and ventrolateral (*lvtp*) perikarya of *vtp*. *amp*–anterior median aggregation of perikarya, *CUP–*cuticle shield, *DC*–dorsal commissure, *DLN*–dorsal longitudinal neurite bundles, *dop–*dorsal aggregation of perikarya, *EP*–epidermis, *EC*–enteral coelom, *LNT*–longitudinal nerve tracts projecting from ventral nerve cord into brain, *LR*–undifferential tentacle lamellae, *lvtp–*ventrolateral perikarya of *vtp*, *NE*–neuropile of the lateral brain lobes, *nep*–peripheral perikarya of the lateral brain lobes, *OBL*–obturacular lobes, *OBC*–obturacular coelom, *OBN*–obturacular neurite bundles, *OBV*–obturacular blood vessels, *pl*–large perikarya, *ps*–small perikarya, *SLN*–supraenteral longitudinal neurite bundles, *TE*–free tentacles (palps), *TEN*–neurite bundles of tentacles (palps), *VSN–*vertical supraenteral neurite bundles, *vtp*–tripartite ventral aggregation of perikarya, *vvtp–*ventral perikarya of *vtp*, *XXL–*pair of prominent bundles of large longitudinal neurites (part of *LNT*).
(TIF)

S3 Fig Middle and posteriormost brain organization of *Riftia*. Comparison of middle (A) and posterior (B) brain sections. Dorsal part of brain occupied by elements of supraesophageal ganglion, whereas ventral part is occupied by subesophageal ganglion elements. Middle brain (A) features two commissures of supraesophageal ganglion (dorsal commissure, *DC*, and supraenteral commissure, *SPC*). Longitudinal nerve tracts (*LNT*) disperse on dorsal side of midbrain (A), whereas they form condensed bundles on ventral side of posterior brain (B). Moreover, they run as circumesophageal connectives surrounding enteral coelom (*EC*). A-B–histological cross sections of 79-mm-long male. Level of section shown in diagram, right lower coner. *CUP–*cuticle shield, *DC*–dorsal commissure, *DLN*–dorsal longitudinal neurite bundles, *dop–*dorsal aggregation of perikarya, *EP*–epidermis, *GA*–giant axons, *EC*–enteral coelom, *LNT*–longitudinal nerve tracts projecting from VNC into brain, *LR*–undifferentiated tentacle lamellae, *lvtp–*ventrolateral perikarya of *vtp*, *NE*–neuropile of lateral brain lobes, *nep*–peripheral perikarya of lateral brain lobes, *OBC*–obturacular coelom, *OBL*–obturacular lobes, *OBN*–obturacular neurites, *OBV*–obturacular blood vessels, *pl*–large perikarya, *pmp*–posterior median perikarya aggregation, *ps*–small perikarya, *SBC*–subenteral commissure, *SPC*–supraenteral commissure, *SLN*–supraenteral longitudinal neurite bundles, *TEN*–neurite bundles of tentacles (palps), *VPN–*posterior vertical median bundles, *vvtp—*ventral perikarya of *vtp*, *VSN*–vertical supraenteral neurite bundles.
(TIF)

S4 Fig Intraepidermal position of the brain. The huge vestimentiferan brain is located in epidermis in anteriormost vestimentum (A). Dorsal furrow between brain lobes encloses obturacules, the anterior appendages of the vestimentum (B-D). Obturacules innervated *via* neurite bundles running along epidermal layer (section 2). A, B–schemes of sagittal and cross sections at levels (1–3) shown in (A). *B*–brain, *CU–*cuticle, *CUP–*cuticle shield, *ECM–*extracellular matrix, *EP*–epidermis, *OBL*–obturacular lobes, *VE*–vestimental process, *VNC–*ventral nerve cord.
(TIF)

S5 Fig Neural elements in the bases of the undifferential tentacle lamellae. Numerous radial tentacle neurite bundles (*TEN*) extend from neuropile of lateral brain lobes to bases of tentacle lamellae (A). Each lamella is a fold of epidermis represented by two layers: thin external lamellae wall (*OEP*) and thick internal lamella wall (*IEP)* containing basiepithelial tentacle neurites (*TEN*). Tentacle lamellae originate at brain periphery of dorsal, lateral and ventrolateral sides of brain (B-D). Dorsalmost lamellae are least differentiated (B). Ventrolateral lamellae extend toward anterior end of tentacle crown (D). They remain undifferentiated over a certain part of their length, and then separate into individual tentacles. A–scheme of neural elements of undifferential tentacle lamellae: perikarya and neurite bundles. B-D–tentacle lamellae bases on dorsal, lateral and ventrolateral sides of brain surface, respectively. *ECM–*extracellular matrix, *EP*–epidermis, *IEP*–epidermis of the internal lamellae wall, *OEP*—epidermis of external lamellae wall, *LR*–undifferentiated tentacle lamellae, *NE*–neuropile of lateral brain lobes, *nep*–peripheral perikarya of lateral brain lobes, *NB*–neurite bundles, *OB–*obturaculum, *pl*–large perikarya, *ps*–small perikarya, *TEN*–neurite bundles of tentacles (palps).
(TIF)

S6 Fig Coelomic channels running through the brain. Three coelomic channels pass through brain: a single enteral coelom (*EC*) and a pair of obturacular channels (*OBC*). The EC encompassing the gut rudiment marks area of peripheral perikarya of lateral brain lobes (*nep*, a.k.a. supraesophageal ganglion) and tripartite aggregation of perikarya (*vtp*, a.k.a. subesophageal ganglion). Obturacular coelomic channels encompassing obturacular blood vessels make an S-like loop in brain tissue. 3D models of *Riftia* brain. A, C, E–peripheral perikarya of lateral brain lobes (*nep*) are on dorsal side of brain (purple). B, D, F–tripartite aggregation of perikarya (*vtp*) is on ventral side and under obturacular and enteral coeloms (blue). View sides shown at right lower corners of each image. Cube side 255 μm. Dashed lines: neural elements under transparent structures. *EC*–enteral coelom, *lvtp–*ventrolateral perikarya of *vtp*, *nep*–peripheral perikarya of lateral brain lobes, *OBC*–obturacular coelom, *pmp*–posterior median perikarya aggregation, *vtp*–tripartite ventral aggregation of perikarya, *vvtp–*ventral perikarya of the *vtp*.
(TIF)

S7 Fig Innervation of neuropile of the lateral brain lobes. Neuropile of lateral brain lobes (*NE*) is the most prominent mass of neurites in supraesophageal ganglion (A-C, A’-C’). Proximately, *NE* connects to longitudinal nerve tracts stemming from ventral nerve cord (*LNT*) (D-H). Dorsal and lateral brain surfaces covered by layer of peripheral perikarya (*nep*, a.k.a. supraesophageal ganglion) (I-J). 3D models of *Riftia* brain. A-C, A'-C'–neuropile of lateral brain lobes (*NE*) associated with longitudinal nerve tracts (*LNT*). D-H–longitudinal nerve tracts projecting from VNC into brain (*LNT*) and giving rise tp prominent bundles of large longitudinal neurites (*XXL*). I-J–peripheral perikarya (*nep*) and neuropile of lateral brain lobes (*NE*). View sides shown at right lower corners of each image. Cube side 255 μm. Dashed lines: neural elements under transparent structures. *DC*–dorsal commissure, *DLN*–dorsal longitudinal bundles, *GA*–giant axons, *EC*–enteral coelom, *LNT*–longitudinal nerve tracts projecting from VNC into brain, *NE*–neuropile of lateral brain lobes, *nep*–peripheral perikarya of lateral brain lobes, *SBC*–subenteral commissure, *SPC*–supraenteral commissure, *vtp*–tripartite ventral aggregation of perikarya, *XXL–*pair of prominent bundles of large longitudinal nerve tracts (part of *LNT*).
(TIF)

S8 Fig 3D-models of anterior neural elements of *Riftia* brain. Anterior median perikarya aggregation (*amp*), with three lobes, is the anteriormost accumulation of big somata (A-C). Three prominent supraenteral longitudinal neurite bundles (*SLN*) connect *amp* with supraenteral commissure (SPC, D-F) and with dorsal commissure (*DC*, F-G). Inverted «ϒ»-shaped vertical supraenteral neurite bundles (*VSN*) connect bundles of *SLN* (A-C). 3D models of *Riftia* brain. A-C–overviews of supraenteral longitudinal neurite bundles (*SLN*) extending from anterior median perikarya aggregation (*amp*); D-E–anterior median perikarya aggregation in association with main cerebral elements: ventral tripartite aggregation (*vtp*), dorsal commissure (*DC*) and supraenteral commissure (*SPC*); F-G–anterior median perikarya aggregation (*amp*) and dorsal commissure (*DC*) in association with obturacular channels (*OBC*). View sides shown at right lower corners of each image. Cube side 255 μm. Dashed lines: neural elements under transparent structures. *amp*–anterior median aggregation of perikarya, *DC*–dorsal commissure, *GA*–giant axons, *EC*–enteral coelom, *OBC*–obturacular coelom, *SBC*–subenteral commissure, *SPC*–supraenteral commissure, *SLN*–supraenteral longitudinal neurite bundles, *VSN–*vertical supraenteral neurite bundles, *vtp*–tripartite ventral aggregation of perikarya.
(TIF)

S9 Fig Obturacular innervation and giant neurons in *Riftia* brain. Dorsalmost side of midbrain shows a pair of dorsal longitudinal bundles (*DLN*, A-C*)* associated with two pairs of obturacular neurite bundles (*OBN*, A-F). Compare innervation pattern of obturacles and tentacles (palps). *OBN* extend from anterior dorsal commissure (*DC*) to bases of obturacles, whereas tentacle neurites are part of brain lobes’ neuropile (*NE*) originating from the longitudinal nerve tracts (*LNT*, D-F). Giant axon (*GA*) in adult vestimentiferans represents fusion of four giant axons: one pair starts from giant perikarya (*gap*) in dorsal commissure (*DC*), another pair from longitudinal nerve tracts (*LNT*, G-L). Note giant perikarya (*gap*) lying in supraesophageal brain part of *Riftia*, like in sabellids. 3D models of *Riftia* brain. A-C–disposition of obturacular neurite bundles (*OBN*) and dorsal longitudinal bundles (*DLN*), D-F–origin of obturacular neurite bundles (*OBN*) from dorsal commissure, and neuropile of lateral brain lobes (*NE*) from longitudinal nerve tracts (*LNT*). G-J–giant axons (*GA*) and position of giant perikarya (*gap*); K-L–position of giant neurons between coelomic channels (*OBC*, *EC*). View sides shown at right lower corners of each image. Cube side 255 μm. Dashed lines: neural elements under transparent structures. *DC*–dorsal commissure, *DLN*–dorsal longitudinal bundles, *GA*–giant axons, *EC*–enteral coelom, *gap–*giant perikarya, *LNT*–longitudinal nerve tracts projecting from *VNC* into brain, *NE*–neuropile of lateral brain lobes, *nep*–peripheral perikarya of lateral brain lobes, *OBC*–obturacular coelom, *OBN*–obturacular neurites, *SPC*–supraenteral commissure, *XXL–*pair of prominent bundles of large longitudinal nerve tracts (part of *LNT*).
(TIF)

S10 Fig Vertical midbrain neurite bundles. Vertical neurite bundles, anterior (*VAN*) and posterior (*VPN*) ones, connect transverse commissures in supraesophageal ganglion (A-D). *VAN*, comprising crossing neurite bundles, extend ventro-dorsally between supraenteral commissure and roots of anterior dorsal commissure (A-C). *VPN* connect supraenteral commissure and posterior dorsal commissure (D). Giant axons (*GA*) following crossing bundles of *VAN* (E-G). 3D models of *Riftia* brain. A-D–anterior (*VAN*) and posterior (*VPN*) vertical median bundles in between other midbrain structures, E-G–giant axons (*GA*) running inside crossing anterior median bundles (*VAN*). View sides shown at right lower corners of each image. Cube side 255 μm. Dashed lines: neural elements under transparent structures. *DC*–dorsal commissure, *GA*–giant axons, *EC*–enteral coelom, *gap–*giant perikarya, *OBC*–obturacular coelom, *SBC*–subenteral commissure, *SLN*–supraenteral longitudinal neurite bundles, *SPC*–supraenteral commissure, *VAN–*anterior vertical median bundles, *VPN–*posterior vertical median bundles.
(TIF)
