# Supplementary figures and images for: The neuroanatomy of the siboglinid Riftia pachyptila highlights sedentarian annelid nervous system evolution
Source: PLoS One. 2018 Dec 13;13(12):e0198271. doi: 10.1371/journal.pone.0198271 (PMC6292602; doi:10.1371/journal.pone.0198271)

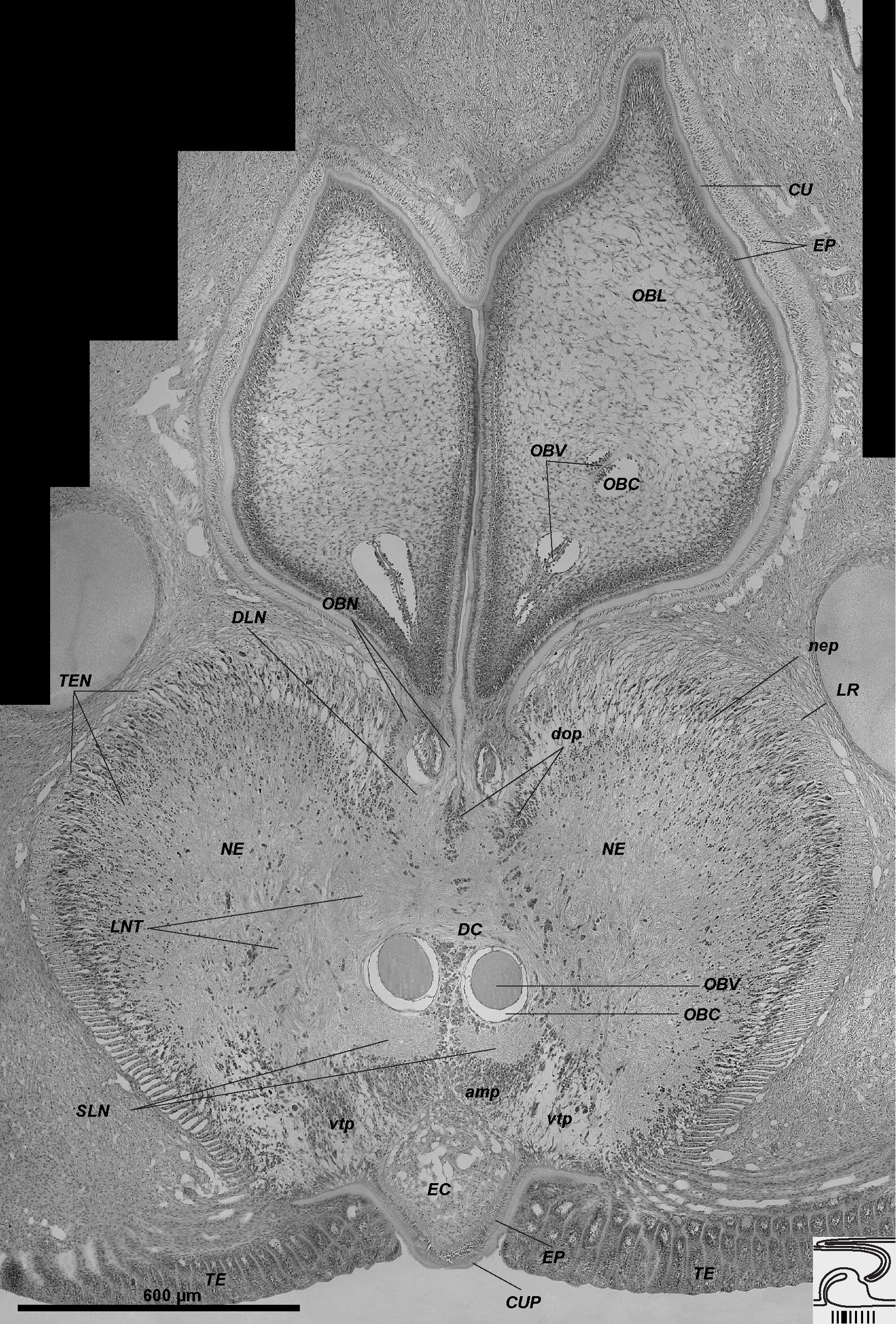

Supplement: S1 Fig — Anteriormost brain contains elements of supraesophageal ganglion (above enteral coelom, EC) and subesophageal ganglia (under EC). In supraesophageal ganglion, the dorsal commissure (DC) connects the longitudinal nerve tracts (LNT) projecting from ventral nerve cord into brain. Obturacular neurite bundles (OBN) enter DC. As a part of the subesophageal ganglion, the tripartite ventral aggregation of perikarya (vtp) expands to anterior part of brain. Level of section shown in diagram, right lower corner. Section level is between sections shown in S2A and S2B Fig. amp–anterior median aggregation of perikarya, CU–cuticle, CUP–cuticle shield, DC–dorsal commissure, DLN–dorsal longitudinal bundles, dop–dorsal aggregation of perikarya, EP–epidermis, EC–enteral coelom, LNT–longitudinal nerve tracts projecting from VNC into brain, LR–undifferentiated tentacle lamellae, NE–neuropile of lateral brain lobes, nep–peripheral perikarya of lateral brain lobes, OBC–obturacular coelom, OBL–obturacular lobes, OBN–obturacular neurite bundles, OBV–obturacular blood vessels, SLN–supraenteral longitudinal neurite bundles, TE–free tentacles, TEN–neurite bundles of tentacles (palps), vtp–tripartite ventral aggregation of perikarya. (TIF) [file pone.0198271.s001.tif]

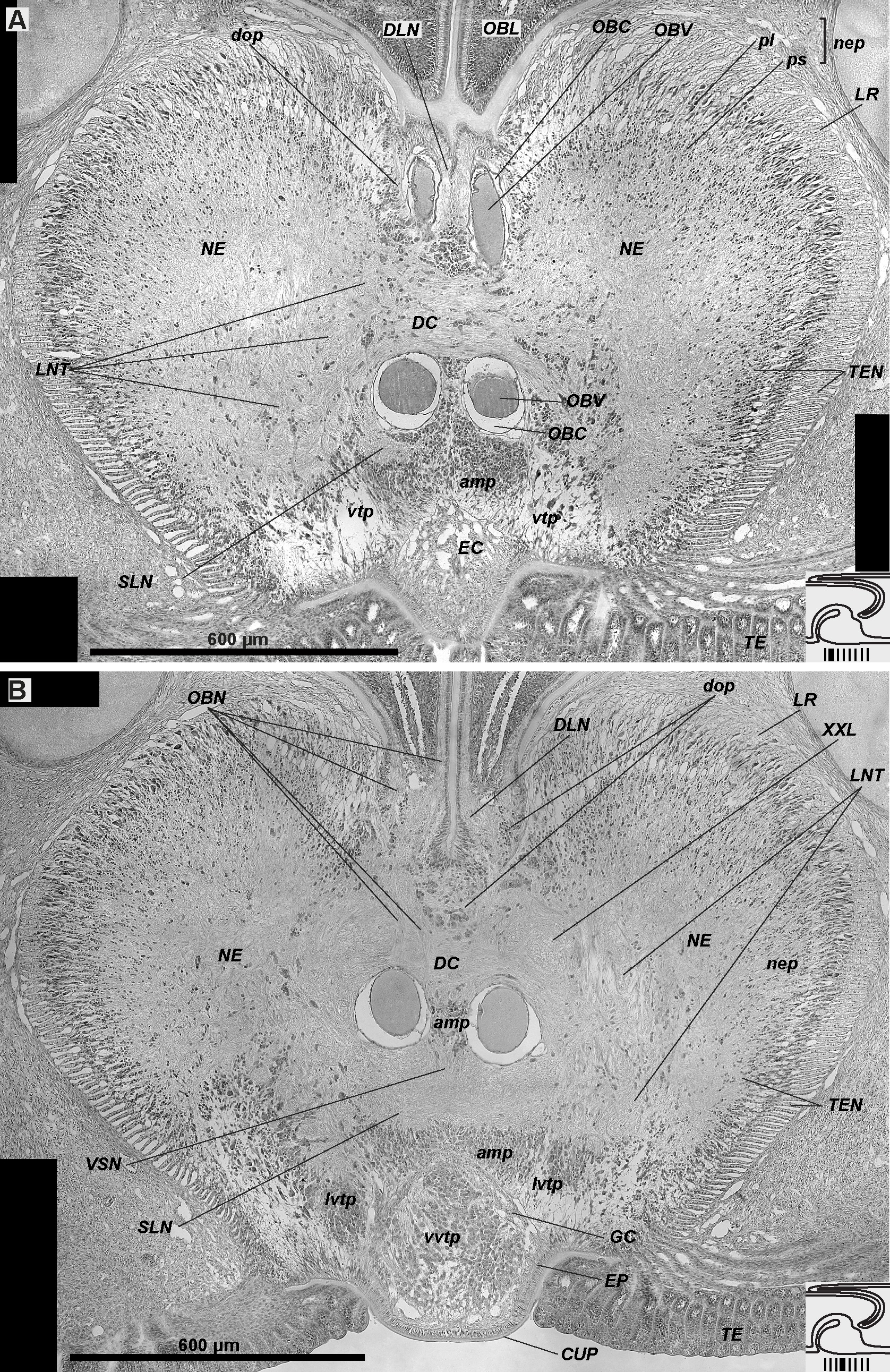

Supplement: S2 Fig — Comparison of anterior (A) and middle (B) brain sections. Anterior brain (A) contains anterior median aggregation of perikarya (amp) and huge areas of neuropile of lateral brain lobes (NE). NE occupies most of dorsal and lateral sides of brain and gives rise to the neurite bundles of tentacles (palps). Huge volume of NE reflects very high number of tentacles (palps) in vestimentiferans, especially in Riftia (up to 335 lamellae pairs, LR). In posterior brain (B), ventral and ventro-lateral sides are occupied by tripartite ventral aggregation of perikarya (vtp) comprising ventral (vvtp) and ventrolateral (lvtp) perikarya of vtp. amp–anterior median aggregation of perikarya, CUP–cuticle shield, DC–dorsal commissure, DLN–dorsal longitudinal neurite bundles, dop–dorsal aggregation of perikarya, EP–epidermis, EC–enteral coelom, LNT–longitudinal nerve tracts projecting from ventral nerve cord into brain, LR–undifferential tentacle lamellae, lvtp–ventrolateral perikarya of vtp, NE–neuropile of the lateral brain lobes, nep–peripheral perikarya of the lateral brain lobes, OBL–obturacular lobes, OBC–obturacular coelom, OBN–obturacular neurite bundles, OBV–obturacular blood vessels, pl–large perikarya, ps–small perikarya, SLN–supraenteral longitudinal neurite bundles, TE–free tentacles (palps), TEN–neurite bundles of tentacles (palps), VSN–vertical supraenteral neurite bundles, vtp–tripartite ventral aggregation of perikarya, vvtp–ventral perikarya of vtp, XXL–pair of prominent bundles of large longitudinal neurites (part of LNT). (TIF) [file pone.0198271.s002.tif]

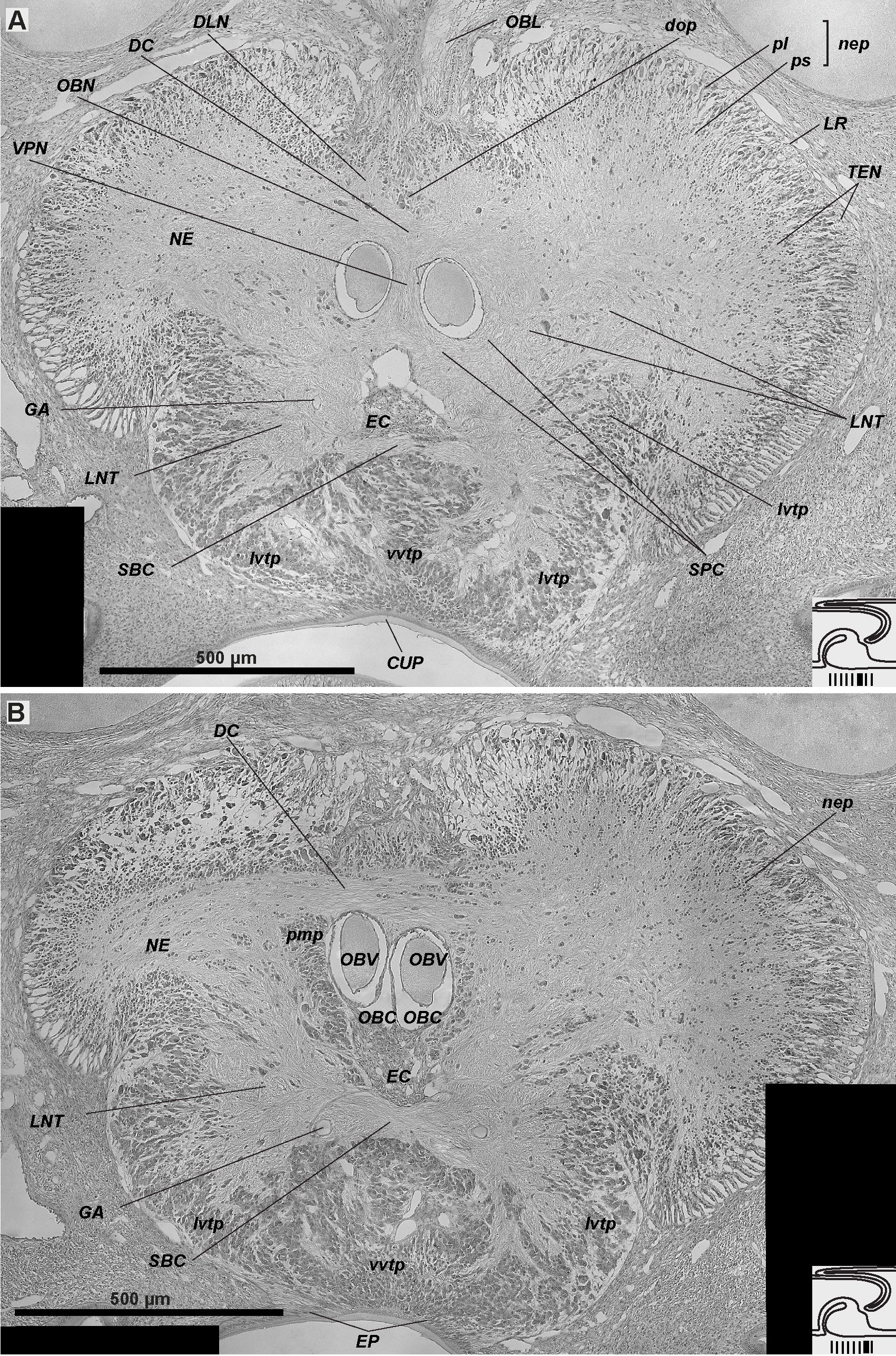

Supplement: S3 Fig — Comparison of middle (A) and posterior (B) brain sections. Dorsal part of brain occupied by elements of supraesophageal ganglion, whereas ventral part is occupied by subesophageal ganglion elements. Middle brain (A) features two commissures of supraesophageal ganglion (dorsal commissure, DC, and supraenteral commissure, SPC). Longitudinal nerve tracts (LNT) disperse on dorsal side of midbrain (A), whereas they form condensed bundles on ventral side of posterior brain (B). Moreover, they run as circumesophageal connectives surrounding enteral coelom (EC). A-B–histological cross sections of 79-mm-long male. Level of section shown in diagram, right lower coner. CUP–cuticle shield, DC–dorsal commissure, DLN–dorsal longitudinal neurite bundles, dop–dorsal aggregation of perikarya, EP–epidermis, GA–giant axons, EC–enteral coelom, LNT–longitudinal nerve tracts projecting from VNC into brain, LR–undifferentiated tentacle lamellae, lvtp–ventrolateral perikarya of vtp, NE–neuropile of lateral brain lobes, nep–peripheral perikarya of lateral brain lobes, OBC–obturacular coelom, OBL–obturacular lobes, OBN–obturacular neurites, OBV–obturacular blood vessels, pl–large perikarya, pmp–posterior median perikarya aggregation, ps–small perikarya, SBC–subenteral commissure, SPC–supraenteral commissure, SLN–supraenteral longitudinal neurite bundles, TEN–neurite bundles of tentacles (palps), VPN–posterior vertical median bundles, vvtp—ventral perikarya of vtp, VSN–vertical supraenteral neurite bundles. (TIF) [file pone.0198271.s003.tif]

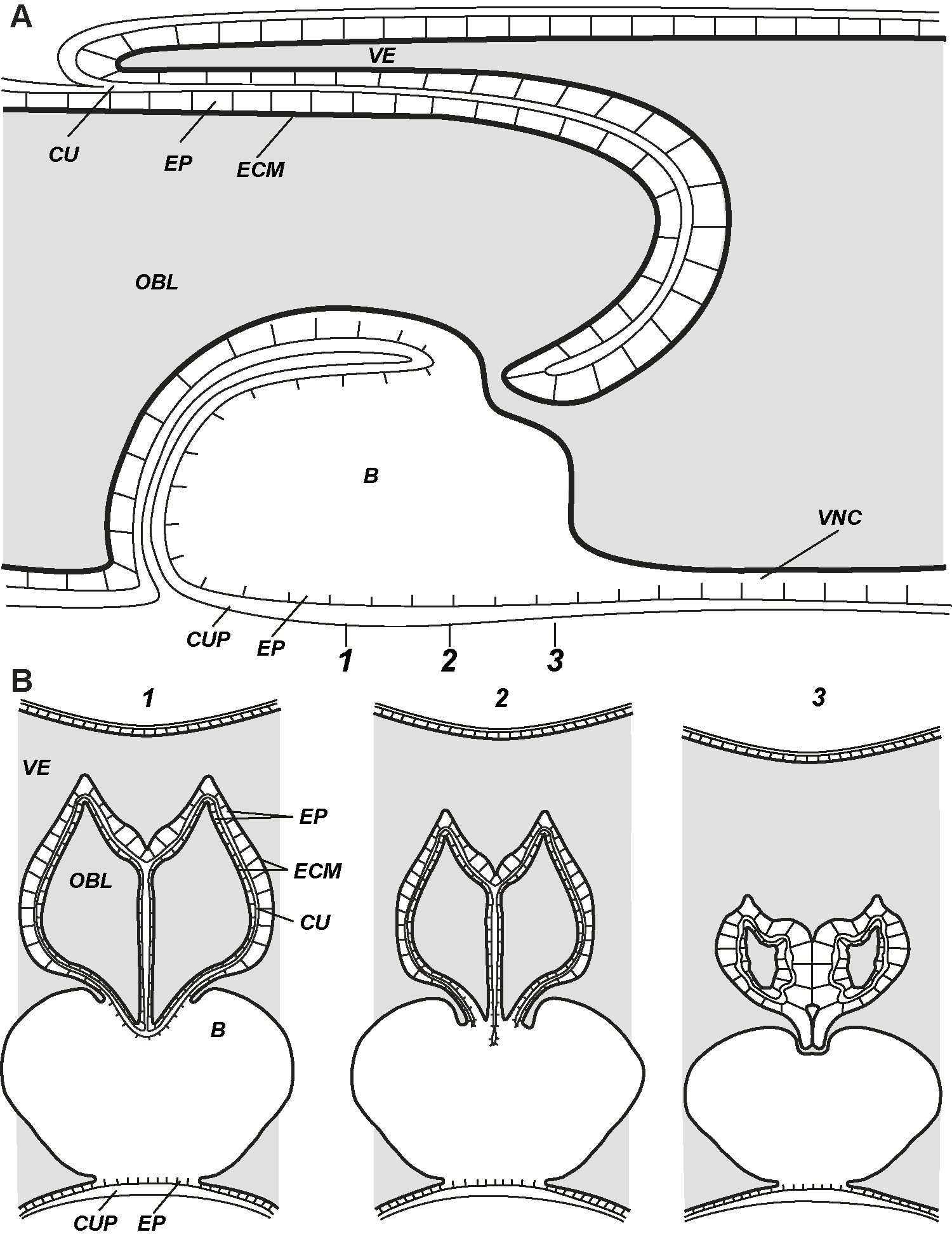

Supplement: S4 Fig — The huge vestimentiferan brain is located in epidermis in anteriormost vestimentum (A). Dorsal furrow between brain lobes encloses obturacules, the anterior appendages of the vestimentum (B-D). Obturacules innervated via neurite bundles running along epidermal layer (section 2). A, B–schemes of sagittal and cross sections at levels (1–3) shown in (A). B–brain, CU–cuticle, CUP–cuticle shield, ECM–extracellular matrix, EP–epidermis, OBL–obturacular lobes, VE–vestimental process, VNC–ventral nerve cord. (TIF) [file pone.0198271.s004.tif]

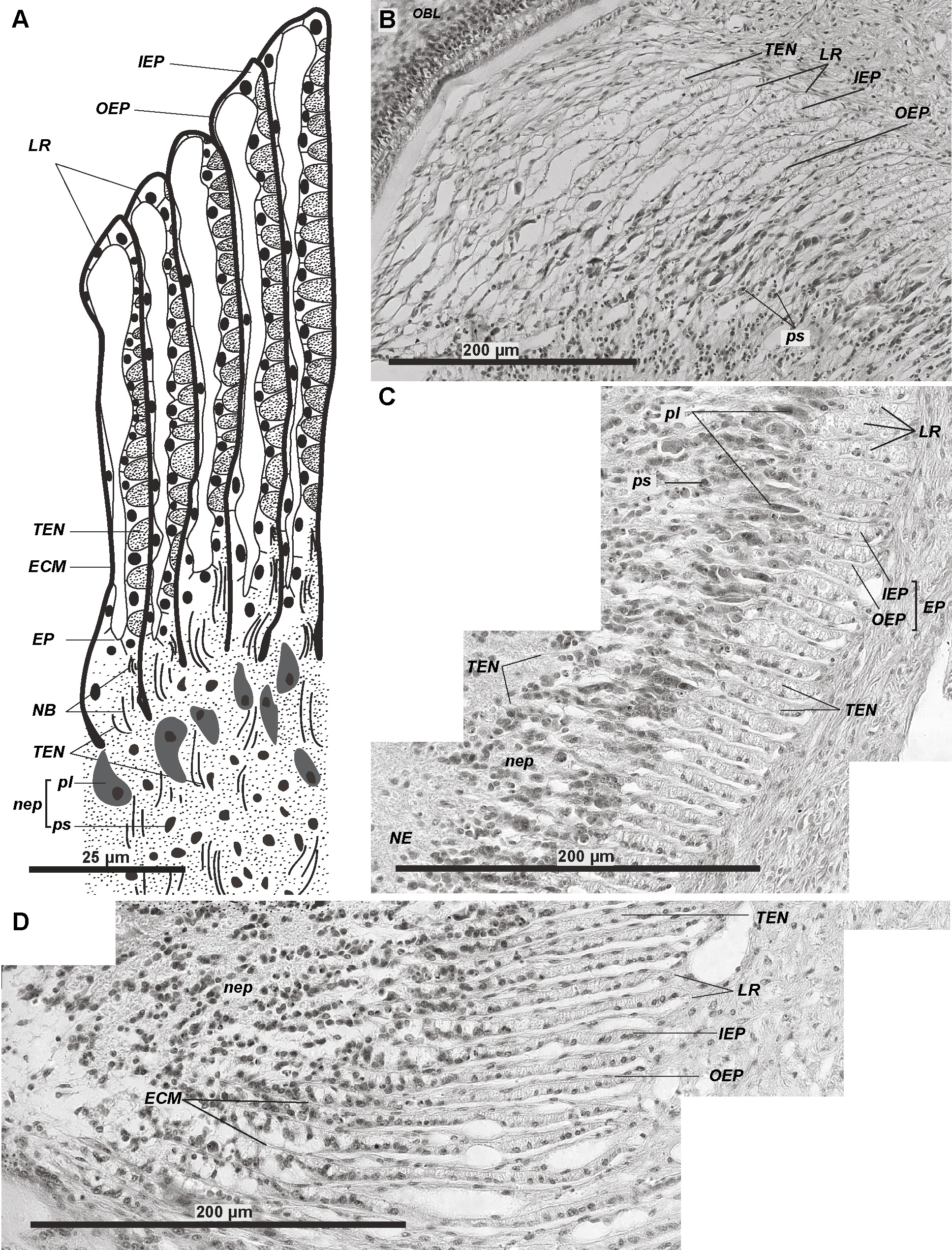

Supplement: S5 Fig — Numerous radial tentacle neurite bundles (TEN) extend from neuropile of lateral brain lobes to bases of tentacle lamellae (A). Each lamella is a fold of epidermis represented by two layers: thin external lamellae wall (OEP) and thick internal lamella wall (IEP) containing basiepithelial tentacle neurites (TEN). Tentacle lamellae originate at brain periphery of dorsal, lateral and ventrolateral sides of brain (B-D). Dorsalmost lamellae are least differentiated (B). Ventrolateral lamellae extend toward anterior end of tentacle crown (D). They remain undifferentiated over a certain part of their length, and then separate into individual tentacles. A–scheme of neural elements of undifferential tentacle lamellae: perikarya and neurite bundles. B-D–tentacle lamellae bases on dorsal, lateral and ventrolateral sides of brain surface, respectively. ECM–extracellular matrix, EP–epidermis, IEP–epidermis of the internal lamellae wall, OEP—epidermis of external lamellae wall, LR–undifferentiated tentacle lamellae, NE–neuropile of lateral brain lobes, nep–peripheral perikarya of lateral brain lobes, NB–neurite bundles, OB–obturaculum, pl–large perikarya, ps–small perikarya, TEN–neurite bundles of tentacles (palps). (TIF) [file pone.0198271.s005.tif]

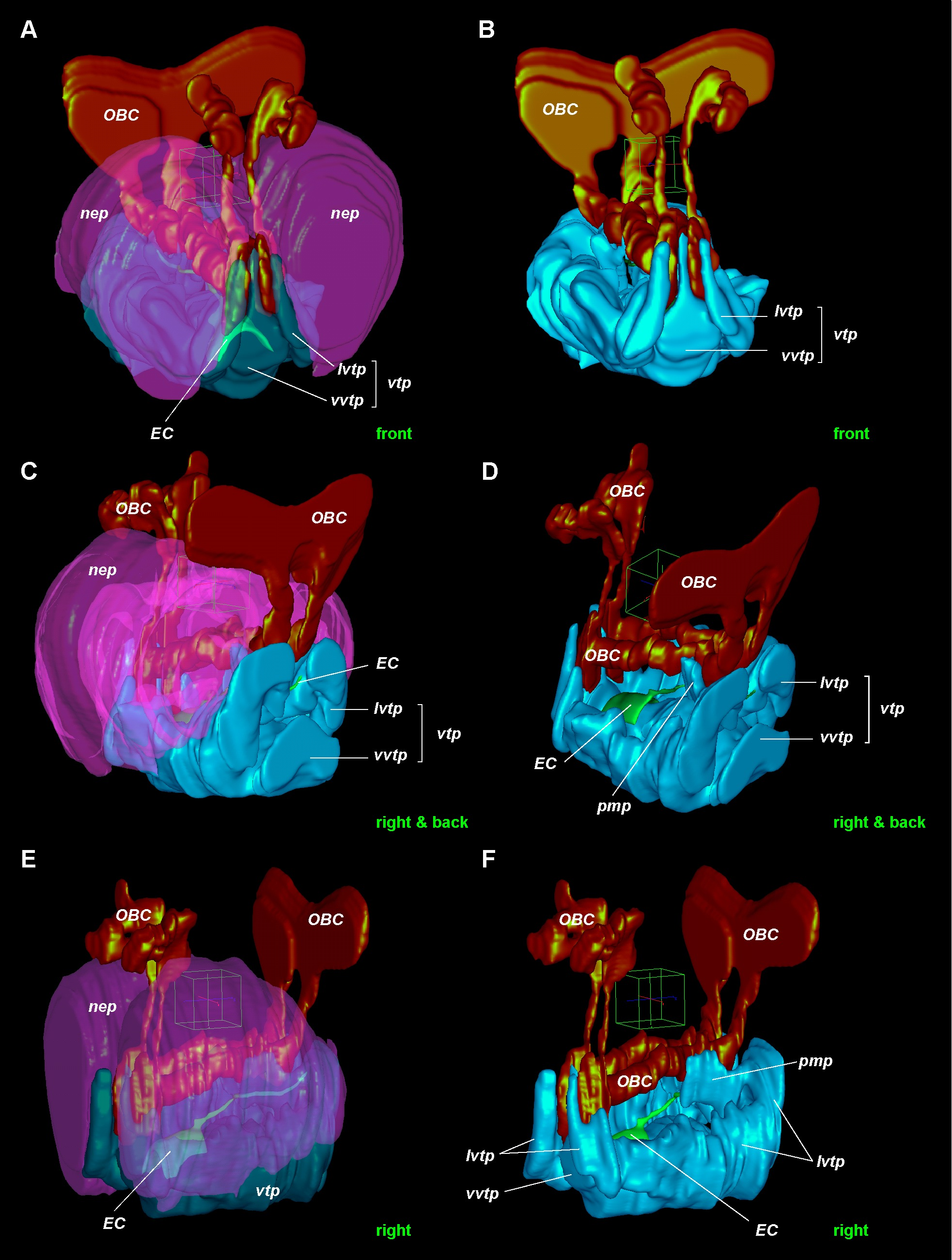

Supplement: S6 Fig — Three coelomic channels pass through brain: a single enteral coelom (EC) and a pair of obturacular channels (OBC). The EC encompassing the gut rudiment marks area of peripheral perikarya of lateral brain lobes (nep, a.k.a. supraesophageal ganglion) and tripartite aggregation of perikarya (vtp, a.k.a. subesophageal ganglion). Obturacular coelomic channels encompassing obturacular blood vessels make an S-like loop in brain tissue. 3D models of Riftia brain. A, C, E–peripheral perikarya of lateral brain lobes (nep) are on dorsal side of brain (purple). B, D, F–tripartite aggregation of perikarya (vtp) is on ventral side and under obturacular and enteral coeloms (blue). View sides shown at right lower corners of each image. Cube side 255 μm. Dashed lines: neural elements under transparent structures. EC–enteral coelom, lvtp–ventrolateral perikarya of vtp, nep–peripheral perikarya of lateral brain lobes, OBC–obturacular coelom, pmp–posterior median perikarya aggregation, vtp–tripartite ventral aggregation of perikarya, vvtp–ventral perikarya of the vtp. (TIF) [file pone.0198271.s006.tif]

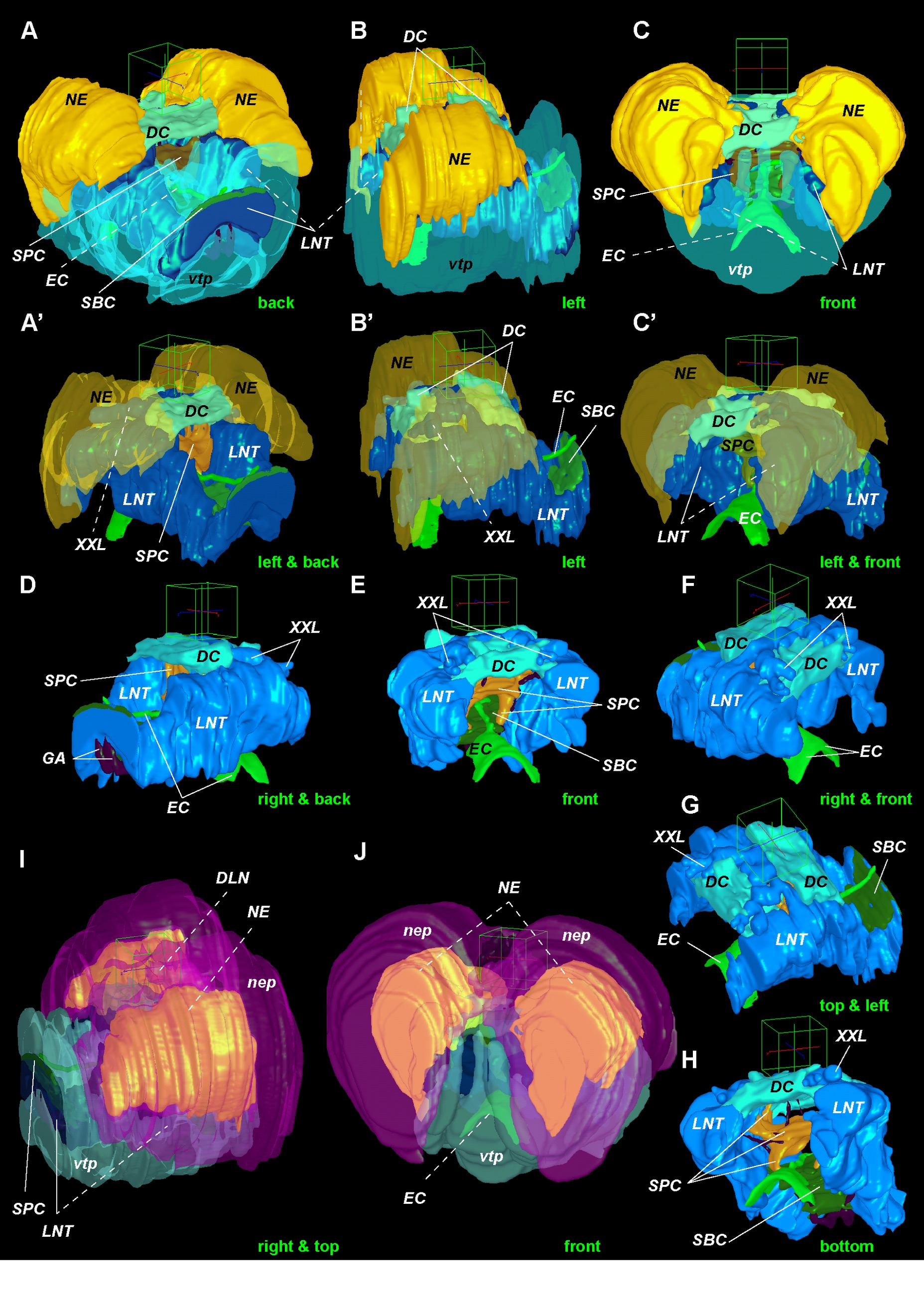

Supplement: S7 Fig — Neuropile of lateral brain lobes (NE) is the most prominent mass of neurites in supraesophageal ganglion (A-C, A’-C’). Proximately, NE connects to longitudinal nerve tracts stemming from ventral nerve cord (LNT) (D-H). Dorsal and lateral brain surfaces covered by layer of peripheral perikarya (nep, a.k.a. supraesophageal ganglion) (I-J). 3D models of Riftia brain. A-C, A'-C'–neuropile of lateral brain lobes (NE) associated with longitudinal nerve tracts (LNT). D-H–longitudinal nerve tracts projecting from VNC into brain (LNT) and giving rise tp prominent bundles of large longitudinal neurites (XXL). I-J–peripheral perikarya (nep) and neuropile of lateral brain lobes (NE). View sides shown at right lower corners of each image. Cube side 255 μm. Dashed lines: neural elements under transparent structures. DC–dorsal commissure, DLN–dorsal longitudinal bundles, GA–giant axons, EC–enteral coelom, LNT–longitudinal nerve tracts projecting from VNC into brain, NE–neuropile of lateral brain lobes, nep–peripheral perikarya of lateral brain lobes, SBC–subenteral commissure, SPC–supraenteral commissure, vtp–tripartite ventral aggregation of perikarya, XXL–pair of prominent bundles of large longitudinal nerve tracts (part of LNT). (TIF) [file pone.0198271.s007.tif]

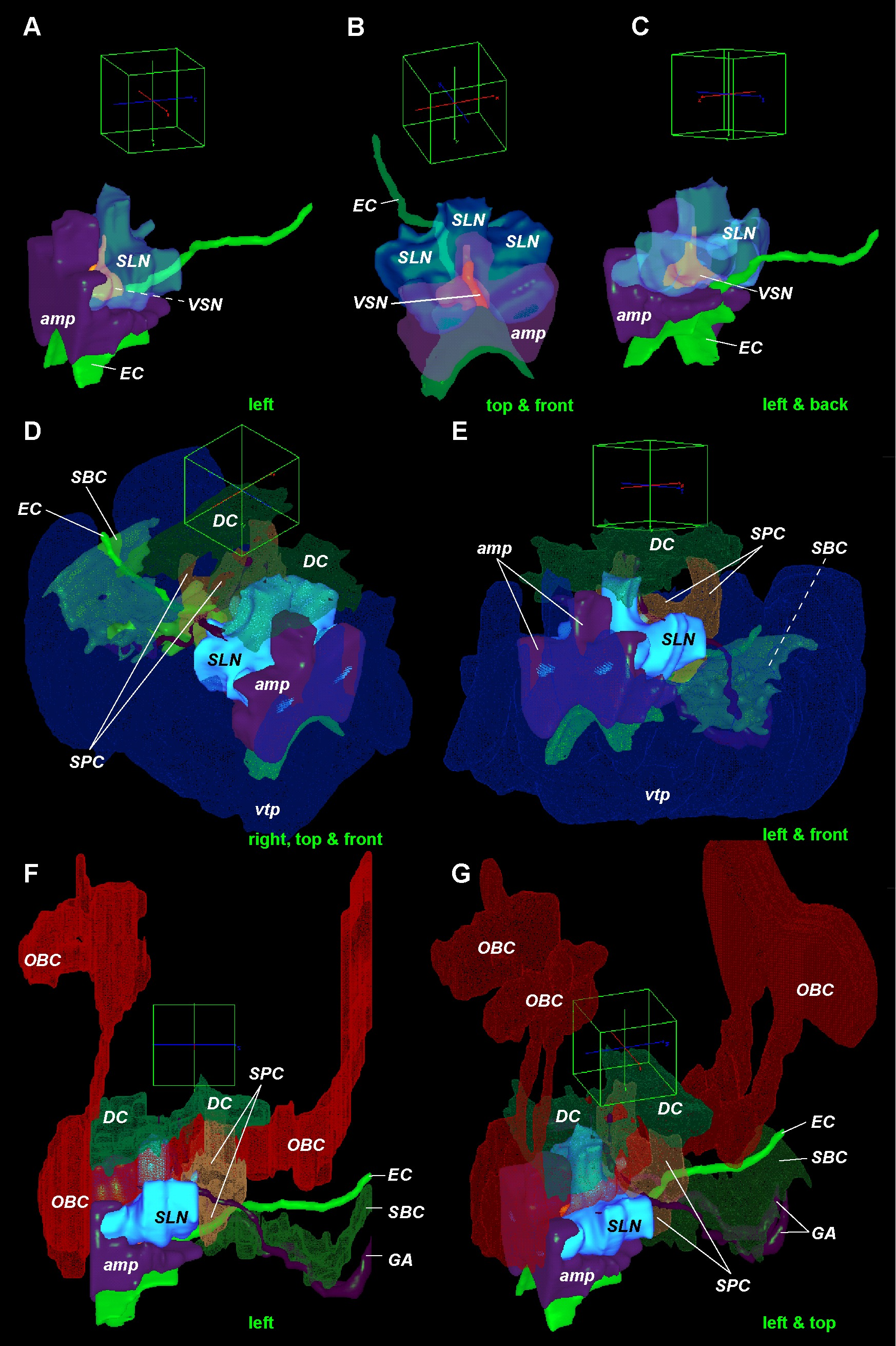

Supplement: S8 Fig — Anterior median perikarya aggregation (amp), with three lobes, is the anteriormost accumulation of big somata (A-C). Three prominent supraenteral longitudinal neurite bundles (SLN) connect amp with supraenteral commissure (SPC, D-F) and with dorsal commissure (DC, F-G). Inverted «ϒ»-shaped vertical supraenteral neurite bundles (VSN) connect bundles of SLN (A-C). 3D models of Riftia brain. A-C–overviews of supraenteral longitudinal neurite bundles (SLN) extending from anterior median perikarya aggregation (amp); D-E–anterior median perikarya aggregation in association with main cerebral elements: ventral tripartite aggregation (vtp), dorsal commissure (DC) and supraenteral commissure (SPC); F-G–anterior median perikarya aggregation (amp) and dorsal commissure (DC) in association with obturacular channels (OBC). View sides shown at right lower corners of each image. Cube side 255 μm. Dashed lines: neural elements under transparent structures. amp–anterior median aggregation of perikarya, DC–dorsal commissure, GA–giant axons, EC–enteral coelom, OBC–obturacular coelom, SBC–subenteral commissure, SPC–supraenteral commissure, SLN–supraenteral longitudinal neurite bundles, VSN–vertical supraenteral neurite bundles, vtp–tripartite ventral aggregation of perikarya. (TIF) [file pone.0198271.s008.tif]

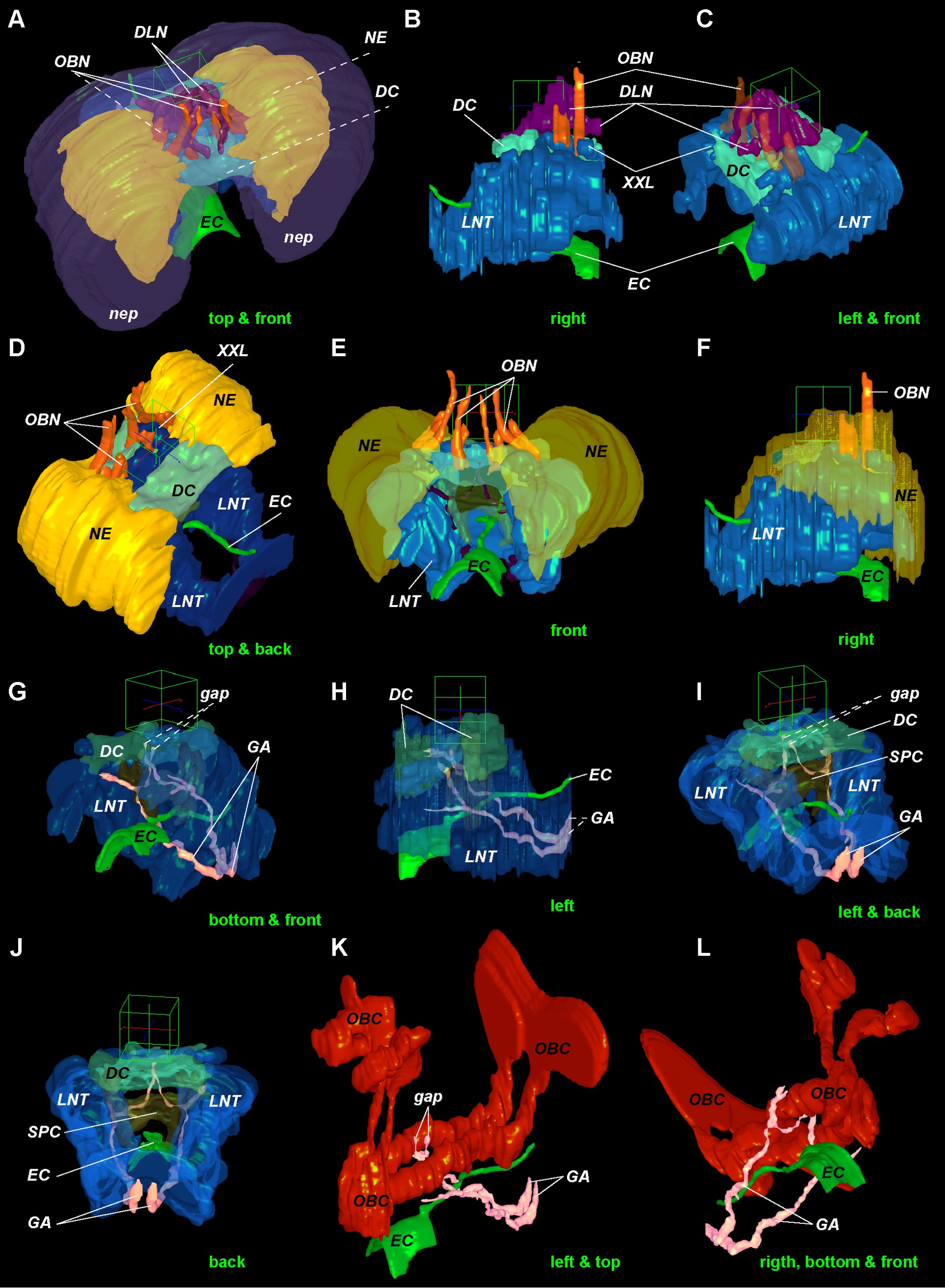

Supplement: S9 Fig — Dorsalmost side of midbrain shows a pair of dorsal longitudinal bundles (DLN, A-C) associated with two pairs of obturacular neurite bundles (OBN, A-F). Compare innervation pattern of obturacles and tentacles (palps). OBN extend from anterior dorsal commissure (DC) to bases of obturacles, whereas tentacle neurites are part of brain lobes’ neuropile (NE) originating from the longitudinal nerve tracts (LNT, D-F). Giant axon (GA) in adult vestimentiferans represents fusion of four giant axons: one pair starts from giant perikarya (gap) in dorsal commissure (DC), another pair from longitudinal nerve tracts (LNT, G-L). Note giant perikarya (gap) lying in supraesophageal brain part of Riftia, like in sabellids. 3D models of Riftia brain. A-C–disposition of obturacular neurite bundles (OBN) and dorsal longitudinal bundles (DLN), D-F–origin of obturacular neurite bundles (OBN) from dorsal commissure, and neuropile of lateral brain lobes (NE) from longitudinal nerve tracts (LNT). G-J–giant axons (GA) and position of giant perikarya (gap); K-L–position of giant neurons between coelomic channels (OBC, EC). View sides shown at right lower corners of each image. Cube side 255 μm. Dashed lines: neural elements under transparent structures. DC–dorsal commissure, DLN–dorsal longitudinal bundles, GA–giant axons, EC–enteral coelom, gap–giant perikarya, LNT–longitudinal nerve tracts projecting from VNC into brain, NE–neuropile of lateral brain lobes, nep–peripheral perikarya of lateral brain lobes, OBC–obturacular coelom, OBN–obturacular neurites, SPC–supraenteral commissure, XXL–pair of prominent bundles of large longitudinal nerve tracts (part of LNT). (TIF) [file pone.0198271.s009.tif]

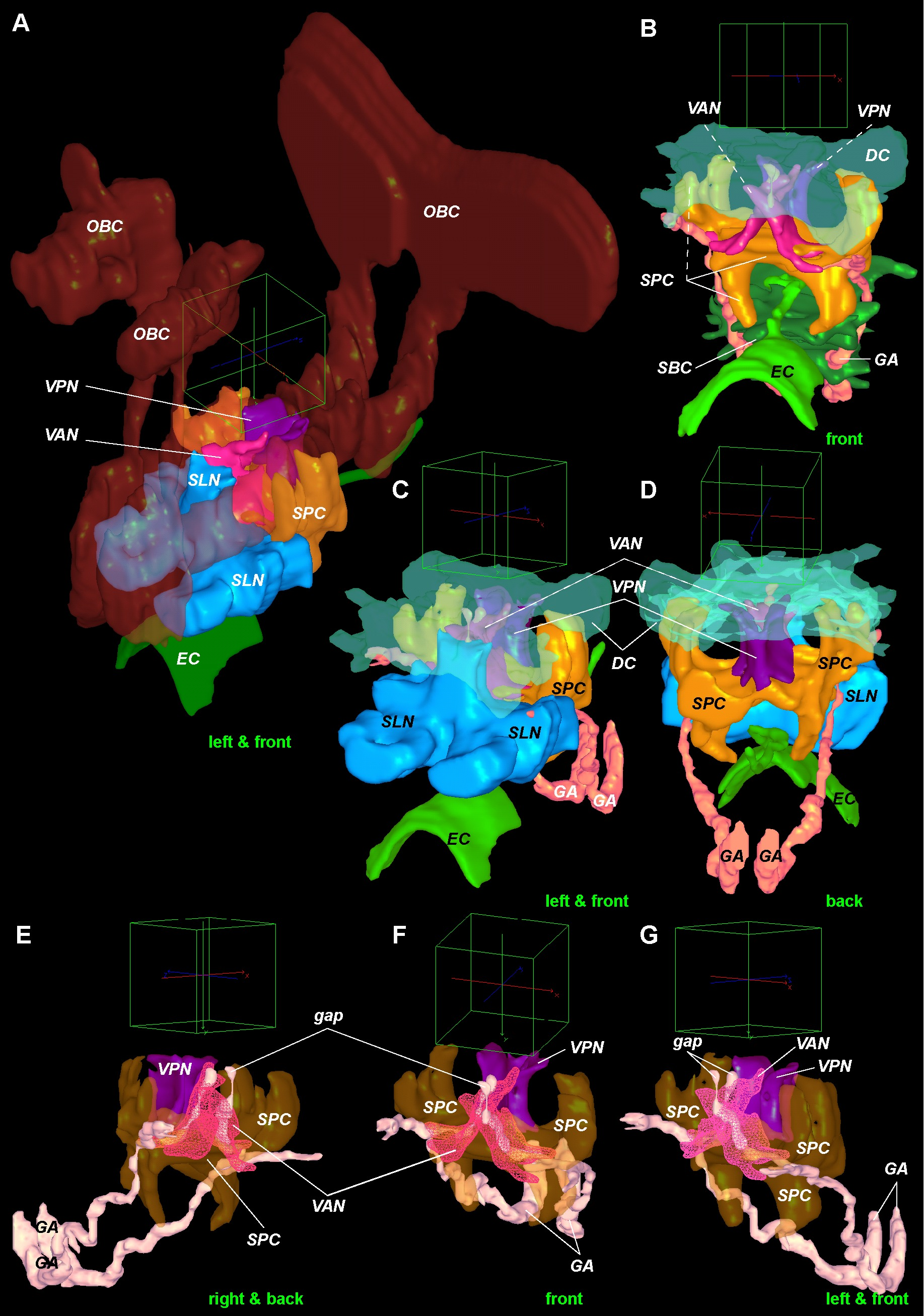

Supplement: S10 Fig — Vertical neurite bundles, anterior (VAN) and posterior (VPN) ones, connect transverse commissures in supraesophageal ganglion (A-D). VAN, comprising crossing neurite bundles, extend ventro-dorsally between supraenteral commissure and roots of anterior dorsal commissure (A-C). VPN connect supraenteral commissure and posterior dorsal commissure (D). Giant axons (GA) following crossing bundles of VAN (E-G). 3D models of Riftia brain. A-D–anterior (VAN) and posterior (VPN) vertical median bundles in between other midbrain structures, E-G–giant axons (GA) running inside crossing anterior median bundles (VAN). View sides shown at right lower corners of each image. Cube side 255 μm. Dashed lines: neural elements under transparent structures. DC–dorsal commissure, GA–giant axons, EC–enteral coelom, gap–giant perikarya, OBC–obturacular coelom, SBC–subenteral commissure, SLN–supraenteral longitudinal neurite bundles, SPC–supraenteral commissure, VAN–anterior vertical median bundles, VPN–posterior vertical median bundles. (TIF) [file pone.0198271.s010.tif]
